# Nutrient and Microbiome-Mediated Plant–Soil Feedback in Domesticated and Wild Andropogoneae: Implications for Agroecosystems

**DOI:** 10.3390/microorganisms11122978

**Published:** 2023-12-13

**Authors:** Amanda Quattrone, Yuguo Yang, Pooja Yadav, Karrie A. Weber, Sabrina E. Russo

**Affiliations:** 1 Complex Biosystems Ph.D. Program, University of Nebraska-Lincoln, Lincoln, NE 68583-0851, USA; 2 School of Biological Sciences, University of Nebraska-Lincoln, Lincoln, NE 68588-0118, USA; yuguo@huskers.unl.edu (Y.Y.);; 3 Center for Plant Science Innovation, University of Nebraska-Lincoln, Lincoln, NE 68583-0705, USA; 4 Department of Earth and Atmospheric Sciences, University of Nebraska-Lincoln, Lincoln, NE 68588-0340, USA; 5 Daugherty Water for Food Institute, University of Nebraska, Lincoln, NE 68588-6203, USA

**Keywords:** plant-microbial interactions, plant-soil feedback, nutrients, wild species, soil domestication, maize, grass

## Abstract

Plants influence the abiotic and biotic environment of the rhizosphere, affecting plant performance through plant–soil feedback (PSF). We compared the strength of nutrient and microbe-mediated PSF and its implications for plant performance in domesticated and wild grasses with a fully crossed greenhouse PSF experiment using four inbred maize genotypes (*Zea mays* ssp. *mays b58*, *B73*-*wt*, *B73*-*rth3*, and *HP301*), teosinte (*Z. mays* ssp. *parviglumis*), and two wild prairie grasses (*Andropogon gerardii* and *Tripsacum dactyloides*) to condition soils for three feedback species (maize *B73*-*wt*, teosinte, *Andropogon gerardii*). We found evidence of negative PSF based on growth, phenotypic traits, and foliar nutrient concentrations for maize *B73*-*wt*, which grew slower in maize-conditioned soil than prairie grass-conditioned soil. In contrast, teosinte and *A. gerardii* showed few consistent feedback responses. Both rhizobiome and nutrient-mediated mechanisms were implicated in PSF. Based on 16S rRNA gene amplicon sequencing, the rhizosphere bacterial community composition differed significantly after conditioning by prairie grass and maize plants, and the final soil nutrients were significantly influenced by conditioning, more so than by the feedback plants. These results suggest PSF-mediated soil domestication in agricultural settings can develop quickly and reduce crop productivity mediated by PSF involving changes to both the soil rhizobiomes and nutrient availability.

## 1. Introduction

Cereal crops make up a large amount of the world’s caloric intake [1]. They require large amounts of water, fertilizer, and other inputs to feed a growing global population [2]. However, growing cereal crops in monoculture for several generations can reduce crop productivity, yield, and soil health through soil domestication [2,3,4,5,6]. More sustainable practices that can replenish soil nutrients and preserve soil health have been introduced into modern agriculture, such as crop rotation, cover crops, and intercropping methods [6,7,8,9,10]. While these practices have increased sustainability, there is still potential to apply ecological frameworks to agricultural systems in order to better understand the underlying mechanisms that result in soil domestication. The framework of plant–soil feedback (PSF) [11] provides a novel lens through which agricultural systems can be understood with respect to the ecological processes operating in them [12]. Plant–soil feedback describes ecological processes in whichplants influence the abiotic and biotic environments in the soil where they grow, and these effects on the soil influence future plant growth in that soil [9,11,13,14].

The mechanisms mediating PSF are related to soil nutrients and microorganisms. Plants take up nutrients via roots, and plant growth is often limited by nutrient availability in the soil [15,16]. Plants respond to changes in soil nutrient availability and other environmental factors through phenotypic plasticity, the magnitude of which often depends on the plant genotype [17,18,19,20,21]. Nutrient-rich soils promote faster plant growth and phenotypes optimized for faster growth, whereas plants in nutrient-poor soils often exhibit greater biomass investment in roots compared to shoots, and they have phenotypes more optimized for nutrient acquisition and conservation [22,23,24]. Plants reduce soil nutrient concentrations through nutrient uptake and growth [25,26,27,28,29], but some of those nutrients are replenished through the decomposition of senesced plant tissues [30]. Variations in nutrient uptake among plant species and genotypes and the consequent reductions in soil nutrient availability [31,32] is one mechanism by which plants may cause PSF.

Other principal mechanisms mediating PSF include the interactions of plants with soil microorganisms. Plants are known to affect the structure of the microbial communities in the soil near and adhering to their roots—this soil region is known as the rhizosphere, and the microbial communities in it are known as the rhizobiome [33,34]. Microbe-mediated PSF effects should vary among plant species and genotypes that have differing effects on soil microbial communities [35,36,37,38,39,40], which can be influenced by differences in the structure and functioning of the root system [41,42,43]. Roots provide a physical surface for associations with microbes [44,45,46,47]. Plant genotypes vary in the structure of their root systems, including how much biomass is allocated to roots versus shoots and organ-level root traits (e.g., root diameter and specific root length) [48,49,50,51]. Roots also modify the chemical environment in the rhizosphere by producing exudates [52,53], which are small molecules, such as amino acids, simple carbohydrates, sugars, and plant hormones produced by the plant and exuded into the soil. Exudates inhibit or promote the growth of microbial taxa, influencing their abundance in the rhizosphere [54,55,56,57,58], and root exudation profiles vary dramatically among plant genotypes and species [59,60,61].

Plant–microbe interactions can have positive or negative effects on the plant through PSF [11]. Microbial communities can influence plant growth along a spectrum of susceptibility and resistance to antagonists such as soil pathogens [42,62,63]. Since many antagonists are host-specific, repeated planting of monocultures in the same soil can cause the accumulation of host-specific soil pathogens [64], with negative effects on plants growing in that soil in the future [65,66]. Plant–soil feedback experiments designed using a phylogenetic gradient have shown that the feedback strength depends on the evolutionary relatedness between the conditioning and feedback species [13,41,67,68]. Microbial associations in the rhizosheath (including the rhizoplane, the region closest to the root, and the rhizosphere) have also been shown to increase nutrient uptake and water use efficiency and increase the growth of cereal crops [32,45,69,70], demonstrating interactive effects between the rhizobiome and nutrient availability. This microbial-mediated feedback can be amplified across generations of plants if the same agricultural practices are used consecutively [41].

Repeated planting of cereal crop monocultures has been shown to cause soil domestication [11], which is likely to be an important mechanism causing negative PSF in agricultural contexts, although it has been less studied [71]. Soil domestication is thought to arise due to reductions in the diversity of types of plant litter and exudate inputs to the soil system, which over time can reduce the diversity and composition of microbial carbon decomposers [30,72], as well as microbial members involved in the nitrogen cycle [32,73]. Most crop land in the Great Plains of North America was derived from native prairie, which originally had high plant diversity growing on deep soils with substantial belowground organic matter content derived from a wide range of plant species [74,75,76]. Intense crop production has led to the domestication of these soils [26,77], with depletion of the available soil nutrients and reduced diversity and compositional variation of soil microorganisms in agricultural versus grassland soils [19,22,71,78]. Ultimately, soil domestication reduces soil health and crop productivity and can also make it more challenging to restore prairies in abandoned agricultural fields due to persistent PSF. Negative PSF is also known to occur in wild grassland systems owing to the accumulation of host-specific pathogens [64]. However, whether cereal crops produce stronger and more negative PSF than wild prairie grass species, as would be suggested by the widespread domestication of agricultural soils, has not been evaluated.

The goal of this study was to quantify variations in the strength of PSF between wild and domesticated grass species in the Andropogoneae tribe of the Poaceae and to evaluate the importance of plant effects on soil nutrient availability and the rhizosphere microbiome in mediating this PSF. We conducted a PSF pot experiment in the greenhouse in which soils were conditioned by two prairie grass species (*Andropogon gerardii* and *Tripsacum dactyloides*) and five maize genotypes (*Zea mays B73*-*wt*, *B73*-*rth3*, *b58*, *HP301*, and *Zea mays* subsp. *parviglumis*) to quantify their effects on the soil microbial community and nutrient concentrations. While plant associations with microbes can involve several domains of life, this study focused on the bacterial and archaeal soil community [79,80,81]. After soil conditioning, three feedback species spanning a gradient of crop to wild prairie grass species (*Zea mays B73*-*wt*, ssp. *parviglumis*, and *Andropogon gerardii*) were sown in the conditioned soils in a fully crossed design to quantify the feedback on plant phenotypes and performance. The plant–soil feedback effect was quantified for each feedback genotype based on differences in the phenotypic traits, growth rates, and effects on soil nutrients among the soil conditioning treatments. We addressed the following questions: (Q1) How is the rhizosphere microbial community structure altered by maize genotypes and wild prairie grass species? How does the PSF depend on conditioning genotype and feedback genotype in terms of (Q2.1) growth, (Q2.2) phenotypic traits, and (Q2.3) effects on soil nutrients? We predicted that the PSF effects would be more negative for maize-conditioned than prairie grass-conditioned soil in that successive generations of maize cultivation would reduce the soil nutrient concentrations and alter the soil microbial diversity and composition in ways that limit beneficial plant–microbe interactions, causing altered phenotypes and reductions in future plant growth.

## 2. Materials and Methods

### 2.1. Study System

The prairie grass conditioning genotypes used in this study provide a gradient of species native to the U.S. Great Plains ecosystems to compare with maize genotypes. *Andropogon gerardii* is the dominant species in tallgrass prairie habitats in the U.S., and it is classified within the Androponeae tribe in the Poaceae [82,83]. *Tripsacum dactyloides* is native to tallgrass prairie ecosystems in the U.S. and is one of the closest extant relatives to modern maize, locally adapted to temperate climates [84,85,86,87]. *Zea mays* subsp. *parviglumis*, which will be referred to by its common name of teosinte in this study, is an ancestral maize lineage that was a progenitor of modern maize genotypes [86,88,89]. *Zea mays B73*, which we will refer to as *B73*-*wt* in this study, is considered one of the first-generation maize hybrids, with greater phenotypic variation than other modern maize genotypes [90,91,92]. The other three *Z. mays* ssp. *mays* genotypes were added to this study due to their documented phenotypic differences from *Z. mays B73*-*wt*. *Z. mays B73*-*rth3* is a naturally derived root hairless mutant of *B73*-*wt* with gene regulatory elements that prevent the elongation of root hairs at the mature root zone, and it has a different root exudate composition compared to the wild-type *Z. mays B73*-*wt* [43,93]. *Z. mays b58* has lower carbohydrate exudates compared to other maize genotypes [61]. *Z. mays HP301* is a variety of popcorn that shows a higher concentration of amino acid exudation than other genotypes [61]. These wild prairie grass species, teosinte, and genotypes of *Z. mays* ssp. *mays* produce a phylogenetic gradient representative of the Andropogoneae tribe in the Poaceae [94,95], which is relevant to maize crops and their current cultivation within U.S. grasslands.

### 2.2. Overview of the Plant–Soil Feedback Experimental Design

**Figure 1:**
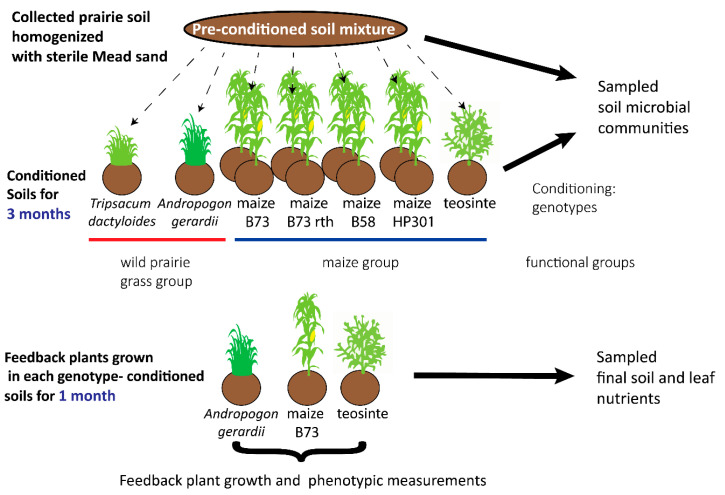
Fully crossed plant–soil feedback (PSF) experimental design. We performed a fully crossed greenhouse plant–soil feedback study involving two phases. An initial (pre-conditioned) soil community was conditioned for three months by seven plant genotypes representing two functional groups (prairie grasses and maize). The grass functional group consisted of two wild prairie grass species—*Andropogon gerardii* (12 pots) and *Tripsacum dactyloides* (12 pots), and the maize functional group consisted of five maize genotypes—*Zea mays B73*-*wt* (12 pots), *B73*-*rth3* (12 pots), *b58* (12 pots), *HP301* (12 pots), and *Zea mays* ssp. *parviglumis* (10 pots). Samples of the pre-conditioned soil, unconditioned soil (not shown), and conditioned soils (after removing the conditioning plants) were analyzed to characterize the microbial (bacterial and archaeal) community. The conditioned soil for each pot was split and potted into three replicate pots, one for each of the three feedback species (*A. gerardii*, *Zea mays B73*-*wt*, and *Zea mays* ssp. *parviglumis*). Since the soil was not homogenized across pots within a conditioning genotype, each feedback plant could be mapped to one of each conditioning replicate. Feedback plants were grown for one month, harvested, and phenotyped for leaf, root, and growth traits. The soil and leaves were analyzed for carbon, nitrogen, phosphorus, potassium, magnesium, and calcium.

**Figure 2:**
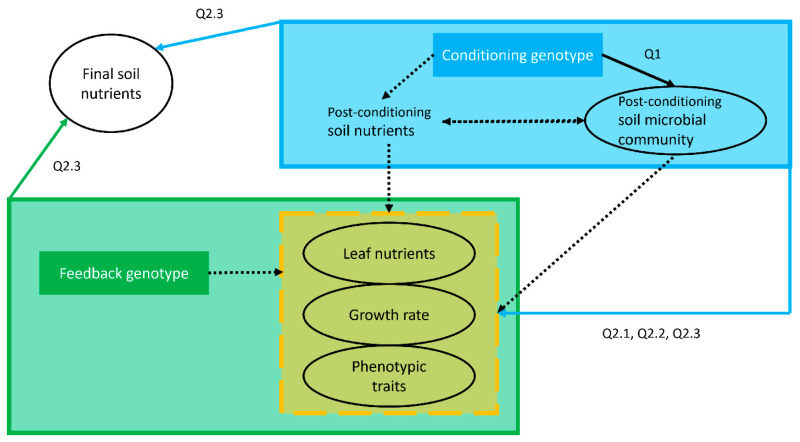
Analysis map for the plant–soil feedback (PSF) experiment. The conceptual diagram shows the PSF mechanisms, the research questions (Q) addressing them (refer to the Introduction for a description of the research questions), and the variables measured. Soil conditioning effects caused by plant conditioning genotypes are indicated by the light blue shaded box, which includes two specific PSF mechanisms examined in this study: effects on soil nutrients and effects on the soil microbial community in the rhizosphere. Phenotypes of the feedback plants grown in the conditioned soil depend both on the genotype of the feedback plant (feedback genotype, green box) and on the effects on soil caused by the conditioning genotype (green arrow). The yellow dashed box indicates measurements of the phenotypes of the feedback plants (Table 1). Solid lines indicate mechanisms specifically addressed in our study, and dashed lines indicate mechanisms that were not addressed by this study. Circles and ellipses indicate variables quantified in this study. The final soil nutrient concentrations are a result of both the effects of soil conditioning (post-conditioning soil nutrients) and the effects of feedback genotypes.

We conducted a PSF pot experiment in the greenhouse (Figure 1 and Figure 2) to compare the strength of PSF between wild and domesticated grass species and the nutrient and microbe–mediated PSF effects. All materials and solutions were autoclave-sterilized, and plants and soils were handled with autoclaved or ethanol-sterilized materials to prevent cross-contamination with microorganisms. The soils were conditioned by seven plant genotypes consisting of two wild prairie grass species—*Andropogon gerardii* (12 pots) and *Tripsacum dactyloides* (12 pots)—and five maize genotypes—*Zea mays B73*-*wt* (12 pots), *B73*-*rth3* (12 pots), *b58* (12 pots), *HP301* (12 pots), and *Zea mays* ssp. *parviglumis* (10 pots). We included unconditioned control pots (3 pots with no plants) to quantify the effects of plant conditioning on the soils. After twelve weeks of conditioning, the rhizosphere soil microbial (defined here as bacterial and archaeal) communities of each pot were sampled and identified using 16S rRNA gene amplicon sequence variants (ASVs). The conditioned soils from each pot were then divided into thirds and transferred directly to new pots for the feedback phase, resulting in three feedback pots (one for each feedback genotype) receiving soil from the same conditioning pot and thereby avoiding pseudoreplication resulting from soil homogenization [96,97]. The feedback genotypes were *Zea mays B73*-*wt* (82 pots), *Z. mays* subsp. *parviglumis* (82 pots), and *A. gerardii* (82 pots). Phenotypic traits, growth rates, foliar nutrient concentrations (Table 1), and final soil nutrient concentrations were sampled and quantified for each biological replicate (feedback pot) after four weeks of growth. The plant soil feedback effect was quantified by comparing the effect of conditioning genotypes within a feedback genotype.

#### 2.2.1. Conditioning Phase: Seedling Growth, Phenotyping, and Collection of Soil Samples

The initial soil community was established using a 60:40 (mass/mass) mixture of naturally sandy soil, which was autoclaved prior to mixing with prairie soil, which served as the soil microbial inoculum. The sandy soil was sterilized by autoclave sterilization with two dry cycles, each of which lasted 100 min at 121 °C temperature and 110 kPa, with a 24 h incubation period in between cycles [98]. The prairie soil serving as the inoculum was collected using sterile instruments from the top 30 cm of a tallgrass prairie, Nine-Mile Prairie (NMP), a remnant prairie owned by the University of Nebraska, located near Lincoln, NE, USA (41.15° N, 96.50° W; elevation 354 m a.s.l.). The prairie soil was sieved to remove any non-soil particles to aid homogenization. The soils were manually homogenized, and a sample of the mixed soil was placed in a sterile 15 mL falcon tube and placed on ice before storage at −80 C until DNA extraction. The remaining mixed soil was allocated equally to sterilized pots (Stuewe and Sons, Inc., Tangent, OR, USA: MT49 pots, 4 in. × 4 in. × 9.5 in., 1.589 L). The soil in the pots was allowed to equilibrate for 5 days in the greenhouse before seedling transplantation. During equilibration, 100 mL of autoclaved ddH_2_O was added daily.

#### 2.2.2. Conditioning Phase: Seedling Growth and Collection of Soil Samples

Seeds of *Zea mays B73*-*wt*, *B73*-*rth3*, *b58*, and *HP301* were obtained from self-pollinated plants grown in a greenhouse. *Zea mays* ssp. *parviglumis*, *A. gerardii*, and *Tripsacum dactyloides* seeds were obtained from open-pollinated plants grown in outside gardens. It is important to note that there is greater genotypic variation among plants of the wild prairie grass species, *A. gerardii* and *T. dactyloides*, due to open pollination compared to the maize genotypes with controlled pollination. Details of the seed sources and genotyping can be found in Supplemental Appendix S1.

Seeds for all genotypes were surface sterilized prior to incubation in 2% Tween 20 in a 1 mM CaCl_2_ solution [99]. The seed coats of *Z. mays* ssp. *Parviglumis* and *T. dactyloides* were physically removed to facilitate germination [100,101]. Seeds were placed in beds of vermiculite in closed seedling trays in the greenhouse and kept moist to promote germination. Throughout the experiment, temperatures in the greenhouse were set at 23.8 °C during daylight and 21.1 °C during nighttime, with full-spectrum lamps supplementing natural light for 12 h each day. One seedling was transplanted into each of the equilibrated pots, watered with ca. 100 mL of ddH_2_O daily and with 50 mL of sterile 25% Hoagland’s solution [102] mixed with sterile 50 mL ddH_2_O once every ten days. Seedlings were grown from transplantation to 12 weeks of age (13 June to 25 October 2019).

Because maize seedlings grow more quickly than the other conditioning genotypes, new seedlings of *Z. mays B73*-*wt*, *B73*-*rth3*, *b58*, and *HP301* were sown in their corresponding pots after six weeks (13 June to 30 July 2019) to continue conditioning. In the greenhouse, the soil was removed from each pot and separated from the roots by manually jostling the soil off into buckets. The soil was re-allocated to pots, and a second set of seeds were treated as described above to obtain seedlings that were then sown in pots conditioned with the corresponding genotype. Seedlings were germinated as described above from transplantation to six weeks (8 August to 25 September 2019).

After the 12 weeks of accumulated soil conditioning, rhizosphere samples (hereafter referred to as soils) were collected from each biological replicate (pot) at harvest [33,34,71]. The seedlings and soil were manually separated to keep the seedlings intact with minimal disturbance of soil adhering to the roots. Rhizosphere samples of 2–3 g of soil were collected proximal to the roots from each pot, transferred into 2 mL microcentrifuge tubes, flash frozen in liquid N_2_, and stored at −78 °C until DNA extraction. The remaining soil volume for each pot was divided into three equivalent volumes and transferred into separate sampling bags (Whirl-Pak, Pleasant Prairie, WI, USA: cat. No. B01489) (ca. 500 g). Approximately 500 mL of conditioned soils were aliquoted to new pots (Stuewe and Sons, Inc., Tangent, OR, USA: TP38 pots, 3 in. × 3 in. × 8 in., 950 mL) and equilibrated for 5 days, adding 30 mL of ddH_2_O daily, prior to transplantation of seedlings for the feedback phase.

#### 2.2.3. Conditioning Phase: DNA Extraction, qPCR, Amplicon Sequencing, and Bioinformatic Analysis

DNA for amplicon sequencing was extracted from soil samples by bead beating in 5% CTAB (cetyltrimethylammonium bromide) followed by phenol:chloroform:isoamyl alcohol (25:24:1) extraction followed by DNA precipitation using 40% polyethylene glycol (PEG) [103,104]. The qPCR copies were determined using the KAPA HiFidelity HotStart Polymerase of the 16S V4 gene regions (515F and 806R primers) for approximately 10× sequencing coverage. Amplicon sequencing targeted the 16S V4 rRNA gene region using the 515F (5′-GTGCCAGCMGCCGCGGTAA) and 806R (5′-GGACTACHVGGGTWTCTAAT) primers and was sequenced with one run of Illumina MiSeq paired-end sequencing (2 × 300 bp) with an expected sequencing depth of 45,000 reads per sample at the University of Minnesota Genomics Center, Minnesota, USA. Across 94 rhizosphere and unconditioned soil samples, including sequencing duplicates of rhizosphere samples collected from three biological replicates (seedlings) and the initial soil sampling triplicates with two sequencing duplicates each, we obtained 4.23 million raw 16S paired gene sequence reads. We analyzed the sequences using DADA2 (v3.10) [105] in R (v3.6.0) to produce an ASV table for the bulk soil and rhizosphere samples from each seedling. The bioinformatic analysis followed the protocol outlined in Quattrone et al. [43].

#### 2.2.4. Feedback Phase: Seedling Growth and Phenotyping

Seeds of the feedback genotypes (*Z. mays* ssp. *Mays B73*-*wt* (maize *B73*-*wt*), *Z. mays* ssp., *parviglumis* (teosinte), and *A. gerardii*) were surface-sterilized, germinated, watered and grown in the same conditions as described in Section 2.2.2. One seedling was transplanted to each pot containing conditioned soil, and the fresh biomass for each seedling was recorded at the time of transplantation. Seedlings were harvested after four weeks of growth post-transplantation (4 November to 11 December 2019). Soil samples (ca. 50 mL) from each pot were collected for quantification of the concentrations of nutrients. The root system was cleaned, and the aboveground portion of the seedling was severed from the root system. Measurements required to estimate growth rates and phenotypic traits (Table 1) were collected for the roots, stems, and leaves of each seedling. Cleaned roots were separated into coarse roots and fine roots with a cutoff of ≤1 mm diameter for fine roots, and they were measured separately for phenotypic analyses. Phenotypic measurements were taken following methods used in our previous studies [43].

#### 2.2.5. Elemental Analysis of Final Soil and Leaf Nutrients

Final soil and leaf samples for each feedback biological replicate were analyzed for six nutrient concentrations (C, N, P, K, Ca, and Mg; Table 1). Leaf concentrations of C and N (%) were estimated using elemental analysis (EA-IRMS) by the UC Davis stable isotope facility (UCDavisSIF). Ground leaf samples were separately prepped with HNO_3_ digestion before the concentrations of P, Ca, K, and Mg (%) were estimated using inductively coupled plasma optical emission spectrometry (SPECTRO ARCOS ICP) by the Agricultural Diagnostic Laboratory of the University of Arkansas (UADA). The final soil samples from each feedback pot were ground and filtered through a 2 mm sieve before analysis for soil C and N concentrations (%) by combustion (Elementar varioMAX CN Cube) and for P, Ca, K, and Mg concentrations (mg nutrient/kg of soil) using ICP-OES analysis after Mehlich 3 extraction (1:10 soil/m^3^ by weight) by the Agricultural Diagnostic Laboratory of the University of Arkansas.

### 2.3. Statistical Analyses

All analyses were performed using R Statistical software (v4.1.2). The ‘microbiome’, ‘phyloseq’, ‘pairwise.t.test’, ‘vegan’, ‘ANCOMBC’, ‘rrBLUP’, ‘lme4’, ‘stats’, ‘car’, ‘ranacapa’, ‘microeco’, and ‘indspecies’ packages were used for statistical analyses [106,107,108,109,110,111,112,113,114,115]. For all tests, statistical significance was assessed at *α* = 0.05.

#### 2.3.1. Q1 Post-Conditioning Soil Microbial Community Structure

We assessed the effects of the conditioning genotype (seven genotypes) on the microbial abundance, richness, diversity, evenness, and composition based on the final ASV table using linear mixed models. Microbial abundance was estimated from quantitative real-time PCR (qPCR) 16S rRNA gene read counts from DNA extracts to estimate the absolute amounts of microbes in this study [116,117]. Measures of alpha diversity for the microbial soil community of each conditioning genotype plant were calculated based on relative abundance. The observed richness was the total number of ASVs; diversity was measured using Shannon’s diversity index and Fisher’s alpha diversity index, and evenness was measured using Simpson’s evenness. We used type III analysis of variance (ANOVA) of separate linear mixed models controlling for conditioning pot replicate for each diversity metric and a fixed conditioning genotype effect.

We assessed the variation in the ASV microbial community composition of the plant functional groups and conditioning genotypes, pre-conditioned soil, and unconditioned soils using a permutational analysis of variance (perMANOVA) in parallel with a principal coordinate analysis (PCoA), relative abundance-weighted (Bray–Curtis) and presence–absence (Jaccard) distance metrics for ordination that were plotted in separate PCoA plots with 95% confidence ellipses for the soil ASV community composition influenced by the conditioning genotype nested within functional groups (prairie grass species versus maize genotype conditioning). Differences in negative control soils and plant-conditioned soils are indicative of plant effects on the community composition. Corresponding perMANOVAs included grass functional groups as a main effect and a nested effect of conditioning genotype within functional groups. Separate plots included pre-conditioned and unconditioned soil communities, which had low biological replication to statistically interpret with multivariate methods. Nested taxonomic bar plots identified taxonomic differences in the microbial community composition at the (1) phylum level, (2) class-level ordered by phylum-level groupings, and (3) genus-level taxonomic classifications ordered by class and family levels for the pre-conditioned, unconditioned, and seven plant-conditioned genotype soil samples. We used an indicator analysis [106] with multiple point-biserial correlation coefficients to assess specific ASVs prevalent and differentiating soil microbial communities for specific conditioning genotypes. To assess ASVs shared across conditioning genotypes, we used intersection transformations to produce summarized UpSet plots [118], resulting in petal plots for (1) soil communities related to all maize-conditioned soils and (2) soil communities related to all plant-conditioned soils for each conditioning genotype.

#### 2.3.2. Q2.1 and 2.2 Phenotypic Traits and Plant Performance of the Feedback Plants

To assess the effects of conditioning genotype on feedback seedling growth, phenotypic traits, and leaf nutrient concentrations (Table 1), we used Type III ANOVAs on separate linear mixed models for each growth rate, phenotypic trait, and foliar nutrient concentration with fixed effects of conditioning genotype, controlling for the conditioning pot replicate and the initial biomass of the seedlings, within pots of each feedback genotype. Pairwise differences between conditioning genotypes were determined after a significant omnibus test with adjustment for post hoc multiple comparisons using FDR within each feedback genotype. We also assessed variations of conditioning genotype nested within functional groups with separate linear mixed-effect models, controlling for the initial biomass of the seedling and the conditioning pot replicate, using Type III ANOVAs.

#### 2.3.3. Q2.3 Variation in Soil Nutrient Concentrations across PSF Phases

We assessed the variation of the influence of feedback genotype, conditioning genotype, and the interaction effects for the final soil nutrient composition using perMANOVA in parallel with non-metric multidimensional scaling (NMDS) plots. Separate NMDS plots for each feedback genotype were created using Gower’s distance metric for the final soil nutrient concentrations of carbon, nitrogen, phosphorus, potassium, calcium, and magnesium with 95% confidence ellipses for the conditioning genotype and functional group effects. Corresponding perMANOVAs tested the main effects of the feedback genotype, the conditioning genotype nested within the functional group, and the interaction effect between the conditioning and feedback genotypes. Similar to the plant performance and phenotypic traits (Section 2.3.2), we assessed the effects of the conditioning genotype on the final soil nutrient concentrations (Table 1) using ANOVAs of separate linear mixed models of the soil conditioning treatment effect (plant-conditioned and unconditioned soils) on each soil nutrient concentration within each feedback genotype, controlling for the initial biomass of the seedlings. Pairwise differences between the conditioning genotypes were determined after a significant omnibus test with adjustment for post hoc multiple comparisons, adjusted using FDR within each feedback genotype.

## 3. Results

After filtering and dereplication, the ASV table used in the statistical analyses had 258,614 total reads across 4850 ASVs. Filtering out spurious taxa, chloroplast hits, and averaging the technical replicates produced 219,601 reads across 3879 ASVs across the conditioning genotypes, soil treatments, and preconditioned soil samples (Table S1). The ASVs were taxonomically classified into one archaeal (Thaumarcheota) and 20 bacterial phyla, spanning 38 classes, 144 families, and 204 genera (315 unique genera, including unclassified genera; if “unclassified,” a genus was nested on a phylogenetic basis into the family and class-level classifications) (Figure S1). Overall, 47% of the genus-level taxa were unclassified, and 15% of the family-level taxa were unclassified. Conditioning genotypes *Zea mays mays b58*, *B73*-*rth3*, and *HP301* produced higher reads, and produced ca. 1.5× more unique ASVs than the other conditioning genotypes (Table S1).

### 3.1. Q1 Shifts in the Soil Microbial Community Structure across Conditioning Genotypes

Soil conditioning caused shifts in the soil microbial community structure in the rhizosphere, with stronger effects in soils conditioned by maize than by prairie grasses and for analyses based on presence–absence rather than relative abundance-weighted metrics (Figure S2). Abundance-weighted metrics showed that the microbial community composition of the pre-conditioned and unconditioned soils were nested within the region defined by the overlap of the maize and prairie grass-conditioned soils (Figure S2A). However, presence–absence metrics showed that the microbial community composition of the maize-conditioned soils shifted away from the prairie grass-conditioned soils, while the microbial communities of the pre-conditioned and unconditioned soils remained nested within the prairie grass-conditioned soils (Figure S2B). Taxonomically, the pre-conditioned microbial community had the lowest relative abundance of Proteobacteria and the highest of Actinobacteria, and the unconditioned control communities had the lowest relative abundances of Verrucomicrobia and Acidobacteria compared to communities in the conditioned soils (Figure S1).

**Figure 3:**
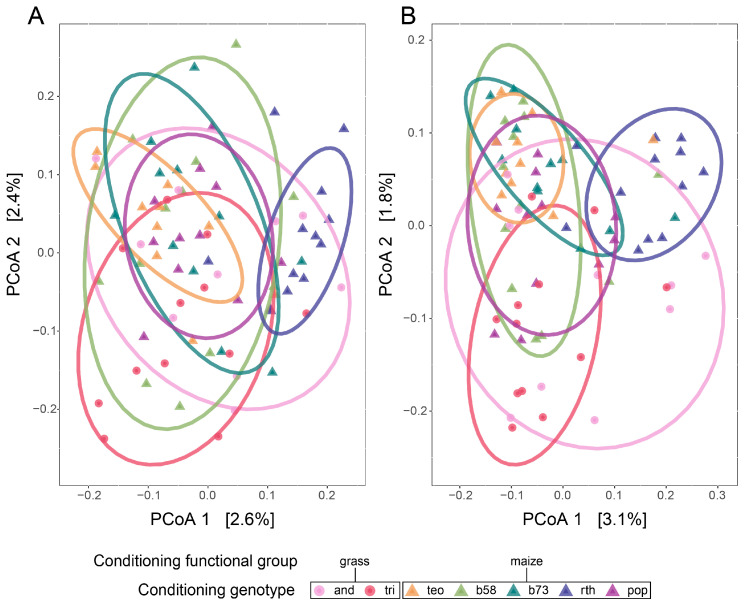
Effects of conditioning genotypes on rhizosphere microbial community structure. Principal coordinate analyses (PCoA) using the (**A**) Bray–Curtis dissimilarity index for abundance-weighted analysis of the amplicon sequence variant (ASV) composition and (**B**) Jaccard presence–absence dissimilarity matrix for the ASV composition. Colors and symbols indicate conditioning genotypes and functional groups, respectively. The prairie grass species (circles) are pink (*Andropogon gerardii*; ‘and’) and red (*Tripsacum dactyloides*; ‘tri’). Maize genotypes are triangles, and the *Z. mays* ssp. *parviglumis* are light orange (teosinte, ‘teo’), while the *Z. mays* ssp. *mays* genotypes are in cooler colors: *b58* is light green, *B73*-*wt* is dark green, *B73*-*rth3* (‘rth’) is dark blue, and *HP301* (‘pop’) is purple. Ellipses represent the 95% confidence ellipse based on the standard deviation around the centroid. The corresponding permutational analysis of variance (perMANOVA) and tests of dispersion can be found in Table 2.

The conditioning genotype nested within the functional group affected the rhizosphere microbial ASV composition more than it affected the alpha diversity metrics and abundance (Table 2 and Table S2; Figure 3 and Figure S1). While no significant differences in abundance nor alpha diversity metrics were shown across conditioning genotypes (Table S2), the ASV composition varied significantly among the conditioning genotypes when nested within functional groups in the abundance-weighted and presence–absence analyses (Table 2; Figure 3B). Prairie grass versus maize-conditioned soils also significantly influenced the variation in the ASV microbial community composition when abundance-weighted or presence–absence metrics were used (Table 2, Figure 3). There was also unique variation independent of the functional groups, as *Z. mays B73*-*rth3* conditioned soils differed from all other conditioned communities in the presence–absence analyses (*p_adj_* ≤ 0.042) (Figure 3A,B). At the phylum level, Patescibacteria and Actinobacteria were more abundant in prairie grass-conditioned soils, whereas Verruccomicrobia was more abundant in maize-conditioned soils than prairie grass-conditioned soils (Figure S1A). The one archaeal phylum, Thaumarcheota, was only present in soils conditioned by *Z. mays parviglumis*, *Z. mays mays B73*-*rth3*, and *Z. mays mays b58* (Figure S1A).

The rhizosphere communities consisted of a greater number of unique ASVs for each conditioning genotype than the shared ASVs between the maize-conditioned or plant-conditioned communities (Table S3; Figure S3). For all plant-conditioned rhizosphere microbial communities, 13 ASVs (0.4%) were shared across all conditioning genotypes, and 12 of the 13 ASVs (92%) mapped to *Candidatus Xiphinematobacter* spp. in phylum Verrucomicrobia while 1 ASV mapped to *Massilia* spp. in Proteobacteria (Figure S3A). For the maize-conditioned rhizosphere microbial communities, 19 ASVs (0.7%) were shared across the maize-conditioning genotypes (Figure S3B). Twelve of the shared ASVs (63%) also mapped to *Candidatus Xiphinematobacter* spp., while one other ASV (5%) mapped to the *Pedosphaeraceae* family in the Verrucomicrobia phylum, two ASVs (10%) mapped to genera in the Proteobacteria phylum (*Massilia* spp. and *Sphingomonas* spp.), three ASVs (16%) mapped to the *Microscillaceae* family in Bacteroidetes, and one ASV (5%) was classified to a family in Actinobacteria (*67*-*14*). While hundreds of rhizosphere microbial ASVs were unique to one conditioning genotype in both analyses (Figure S3), the indicator analysis found only a few ASVs, which may drive community compositional differences for each conditioning genotype (Table S3). Communities conditioned by *A. gerardii* plants had 20 indicator ASVs spanning across 13 genera in nine families within four phyla (Actinobacteria, Bacteroidetes, Patescibacteria, and Proteobacteria), including four ASVs mapped to *Streptomyces* spp. and three mapped to *Actinocatenispora* spp. in Actinobacteria. Communities conditioned by *T. dactyloides* plants had four indicator ASVs spanning across four genera in four families (Dongiaceae, Saccharimonadaceae, Sphingomonadaceae, and Streptomycetaceae) within three phyla (Actinobacteria, Patescibacteria, and Proteobacteria). Communities conditioned by teosinte plants had 14 indicator ASVs mapped to 14 genera in 14 families within nine phyla (Actinobacteria, Acidobacteria, Bacteroidetes, Chloroflexi, Fibrobacteres, Gemmatimonadetes, Planctomycetes, Proteobacteria, and Verrucomicrobia). Communities conditioned by maize *B73*-*rth3* plants had nine indicator ASVs spanning across four genera in three families (Xiphinematobacteraceae, Pedosphaeraceae, and Gemmatimonadaceae) within two phyla (Gemmatimonadetes and Verrucomicrobia), including six ASVs mapped to *Candidatus Xiphinematobacter* spp. in Verrucomicrobia. Communities conditioned by maize *HP301* plants had five indicator ASVs spanning across five genera in four families (Burkholderiaceae, Gemmatimonadaceae, Intrasporangiaceae, and Nocardioidaceae) within three phyla (Actinobacteria, Gemmatimonadetes, and Proteobacteria). Communities conditioned by maize *b58* had the lowest number of indicator ASVs (2) mapped to *Ralstonia* spp. in Burkholderiaceae (Proteobacteria) and a sequence for the Microscillaceae family (Bacteroidetes).

### 3.2. Q2.1 Variation of Feedback Plant Performance across Conditioning Genotypes

**Figure 4:**
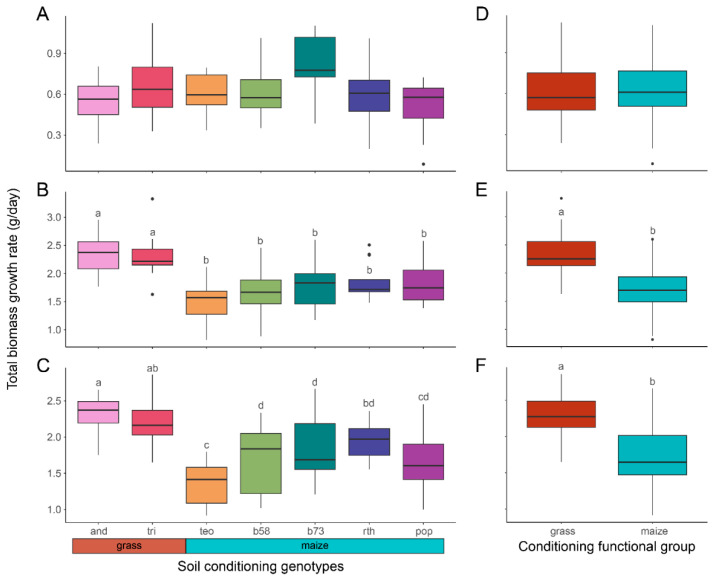
Variations in the conditioning genotype and functional group effects of the plant performance of each feedback genotype. Total biomass growth rates were assessed for the conditioning genotype effect (**A**–**C**) and the conditioning genotype nested within the conditioning functional groups (prairie grass species versus maize genotypes) (**D**–**F**) using type III analysis of variance (ANOVAs) of separate linear mixed models for *A. gerardii* (**A**,**D**), maize *B73*-*wt* (**B**,**E**), and teosinte feedback plants (**C**,**F**). Colors indicate conditioning genotypes and conditioning functional groups. The conditioning functional groups in the key for parts (**A**–**C**) and the boxplots in parts (**D**–**F**) were colored so the prairie grass spp. group (*A. gerardii* and *T. dactyloides*) coordinated with a red–orange hue, while the maize genotypes groups (*Z. mays* ssp., *parviglumis*, *Z. mays* ssp. *mays b58*, *B73*-*wt*, *B73*-*rth3*, and *HP301*) coordinated with a light blue hue. The prairie grass species are pink (*Andropogon gerardii*; ‘and’) and red (*Tripsacum dactyloides*; ‘tri’). Maize genotypes of the *Z. mays* ssp. *parviglumis* are light orange (teosinte, ‘teo’) and the *Z. mays* ssp. *mays* genotypes are in cooler colors: *b58* is light green, *B73*-*wt* is dark green, *B73*-*rth3* (‘rth’) is dark blue, and *HP301* (‘pop’) is purple. The conditioning functional groups in parts (**D**–**F**) were colored so the prairie grass spp. group (*A. gerardii* and *T. dactyloides*) coordinated with a red–orange hue while the maize genotypes groups (*Z. mays* ssp., *parviglumis*, *Z. mays* ssp. *mays b58*, *B73*-*wt*, *B73*-*rth3*, and *HP301*) coordinated with a light blue hue. Lowercase letters indicate significant differences between conditioning genotypes or nested functional groups. The significance between conditioning genotypes (**A**–**C**) was deduced from post hoc pairwise comparisons using Benjamini–Hochberg correction. Refer to Table 3 for the omnibus tests.

The conditioning genotype effects on the biomass growth rate differed among the feedback species (Table 3; Figure 4). *A. gerardii* feedback plants had no significant differences in growth between the conditioning genotypes nor functional groups (Table 3; Figure 4A,D), whereas maize *B73*-*wt* and teosinte feedback plants exhibited significant differences between prairie grass-conditioned and maize-conditioned soils (Table 3; Figure 4B,C,E,F). Maize *B73*-*wt* feedback plants grown in soils conditioned by either prairie grass species consistently showed significantly faster growth rates compared to plants grown in soils conditioned by maize genotypes (Figure 4B). Teosinte feedback plants in soils conditioned by *A. gerardii* also grew faster compared to any maize-conditioned soils, and plants in soils conditioned by *T. dactyloides* grew significantly faster compared to all maize-conditioned soils, except for conditioning by *Z. mays B73*-*rth3* (Figure 4C). Additionally, self-conditioned teosinte plants had the slowest growth compared to teosinte plants conditioned in most non-self-conditioned soils, except for conditioning by *Z. mays HP301* (Figure 4C). Overall, maize and teosinte feedback plants performed better in soils conditioned by prairie grass species compared to maize-conditioned soils (*p* ≤ 0.019; Figure 4E,F).

### 3.3. Q2.2 Variation in Phenotypic Traits and Leaf Nutrient Concentrations of Feedback Plants across the Conditioning Genotype

**Figure 5:**
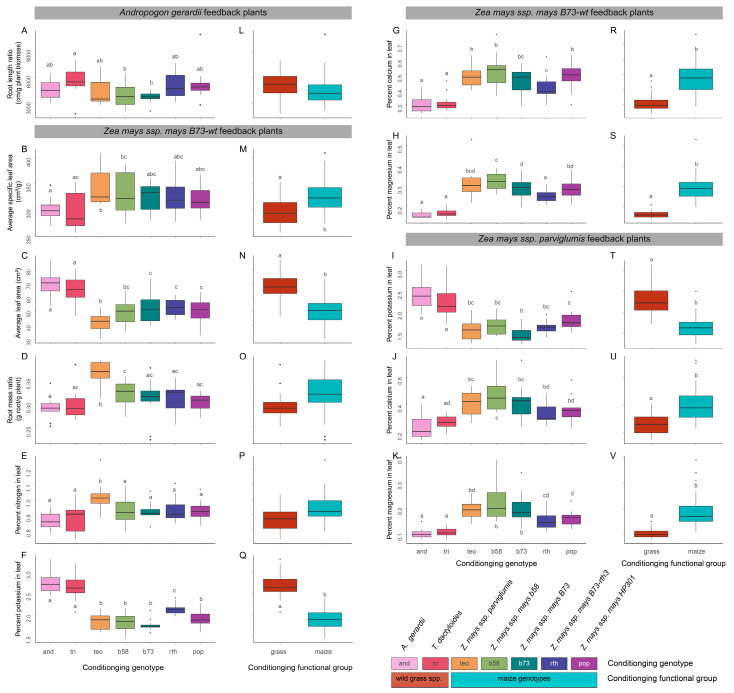
Variation in phenotypic traits and foliar nutrient concentrations of feedback plants among the conditioning genotypes. Phenotypic traits and leaf nutrient concentrations were assessed for a conditioning genotype effect (**A**–**K**) and a conditioning genotype nested within conditioning functional groups (prairie grass species versus maize genotypes) (**L**–**V**) using type III analysis of variance (ANOVAs) of separate linear mixed models for phenotypic measurements that significantly differed within each feedback genotype, including the root length ratio in *A. gerardii* feedback plants (**A**,**L**), average specific leaf area (**B**,**M**), average leaf area (**C**,**N**), root mass ratio (**D**,**O**), leaf nitrogen (**E**,**P**), leaf potassium (**F**,**Q**), leaf calcium (**G**,**R**), and leaf magnesium (**H**,**S**) in maize *B73*-*wt* feedback plants(**B**–**H**,**M**–**S**), along with leaf potassium (**I**,**T**), leaf calcium (**J**,**U**), and leaf magnesium (**K**,**V**) in teosinte feedback plants (**I**–**K**,**T**–**V**). Differences between conditioning genotypes in parts (**A**–**K**) were determined based on the post hoc pairwise comparisons using Benjamini–Hochberg correction between the conditioning genotypes for each significant (*p* < 0.05) phenotypic trait in the type III ANOVA omnibus tests (Table 3) across the soil conditioning genotypes. Colors indicate the conditioning genotypes in (**A**–**K**) and the conditioning functional groups in the (**L**–**V**) plots. See the in-figure legend for the correspondence of the colors with the plant genotypes and functional groups. The significance between the conditioning genotypes (**A**–**K**) was determined from post hoc pairwise comparisons using Benjamini–Hochberg correction. Lowercase letters within each figure indicate significant (*p* ≤ 0.05) pairwise comparisons between groups after correction for multiple comparisons. Refer to Table 3 for the omnibus tests.

The phenotypes and foliar nutrient concentrations of the feedback plants varied significantly due to soil conditioning, but the patterns of variation in soil conditioning genotype and functional groups differed among the feedback plant species for each phenotypic trait (Table 3; Figure 5). *A. gerardii* feedback plants in soils conditioned by *T. dactyloides* showed a significantly higher RLR than soils conditioned by *Z. mays b58* or *B73*-*wt* (*p_adj_* ≤ 0.047 and 0.055, respectively) (Table 3, Figure 5A). However, comparisons between conditioning functional groups (the prairie grass and maize conditioning groups) did not show any significant effects on feedback RLR or other phenotypic traits for the *A. gerardii* feedback plants (Table 3, Figure 5L). Maize *B73*-*wt* feedback plants had a significant conditioning genotype effect on the SLA, LA, and RMR, and varying strengths of functional group effects (Table 3, Figure 5B–D,M–O). Maize *B73*-*wt* feedback plants in soils conditioned by teosinte had the highest RMR (*p_adj_* < 0.003) and a higher SLA (*p_adj_* ≤ 0.030) compared to soils conditioned by prairie grass species (Table 3; Figure 5B,D). Maize *B73*-*wt* feedback planted in soils conditioned by *A. gerardii* also showed a significantly lower RMR than plants conditioned by maize *b58* (*p_adj_* = 0.034) (Figure 5D). In contrast, the average leaf area for maize *B73*-*wt* feedback plants was significantly higher in prairie grass-conditioned soils compared to maize genotypes, with the lowest leaf area in soils conditioned by teosinte (*p_adj_* = 0.091 with *b58* and *p_adj_* ≤ 0.041) (Table 3; Figure 5C). Maize *B73*-*wt* feedback plants in soils conditioned by the prairie grass functional group also had a significantly lower SLA (Figure 5M; ANOVA, type III: *F_1,4_* = 9.96, *p =* 0.030) and a higher average leaf area (Figure 5N; ANOVA, type III: *F_1,5_* = 38.33, *p* = 0.002) than the maize-conditioned soil group. However, the maize *B73*-*wt* plants did not have a significant functional group effect on the RMR (Figure 5O). Overall, the phenotypic traits of maize feedback plants were influenced by the conditioning genotype and functional group, whereas plant conditioning had little to no influence on the phenotypic traits of the *A. gerardii* feedback plants. Teosinte feedback plants did not show differences in structural phenotypes but exhibited differences in foliar nutrient incorporation between conditioning genotypes.

The feedback plant foliar nutrient concentrations generally differed in the unconditioned soils compared to the plant-conditioned soils (Table 3; Figure S4). While the *A. gerardii* feedback plants had no significant differences in foliar nutrient concentrations, the maize *B73*-*wt* and teosinte feedback plants showed similar feedback effects of the foliar nutrient concentrations for all but nitrogen (Table 3; Figure 5 and Figure S4). Feedback genotypes grown in unconditioned soils had the highest foliar nitrogen, phosphorus, and potassium compared to plant-conditioned soils (Figure S4B–D,H–J,N–P) with no significant differences in foliar carbon (Figure S4A,G,M). The foliar calcium concentration for maize feedback plants in unconditioned soils was more similar to prairie grass-conditioned soils (Figure S4K,Q), while the foliar magnesium concentration in unconditioned soils was more similar to maize-conditioned soils (Figure S4L,R). Within the plant-conditioned soils, maize *B73*-*wt* feedback plants had the highest percent nitrogen in leaves when grown in teosinte-conditioned soils (*p_adj_* ≤ 0.042) (Figure 5E), but no other conditioning genotypes nor functional groups showed significant differences (Figure 5E,P). Both maize *B73*-*wt* and teosinte feedback plants had significantly higher foliar potassium in soils conditioned by prairie grass species compared to maize-conditioned soils (functional group) (*p* < 0.001) (Figure 5Q,T). Within the maize-conditioned soils, maize *B73*-*wt* feedback plants had higher foliar potassium in *B73*-*rth3*-conditioned soils (*p_adj_* ≤ 0.021) (Figure 5F), and teosinte feedback plants had lower foliar potassium in maize *B73*-*wt*-conditioned soils than maize *HP301* (*p_adj_* = 0.003) (Figure 5I). In contrast, the foliar calcium and magnesium concentrations were significantly lower in prairie grass-conditioned soils compared to maize-conditioned soils for both maize *B73*-*wt* and teosinte feedback plants (*p* ≤ 0.026) (Figure 5R,S,U,V), with maize *b58*-conditioned soils showing larger foliar calcium and magnesium concentrations (*p_adj_* ≤ 0.004), with varying significances between soils conditioned by teosinte, maize *B73*-*wt*, and *HP301* (Figure 5G,H,J,K). Overall, maize feedback plants differed in leaf nutrient incorporation between the conditioning functional groups.

### 3.4. Q2.3 Effects of Conditioning and Feedback Genotypes on Nutrients

**Figure 6:**
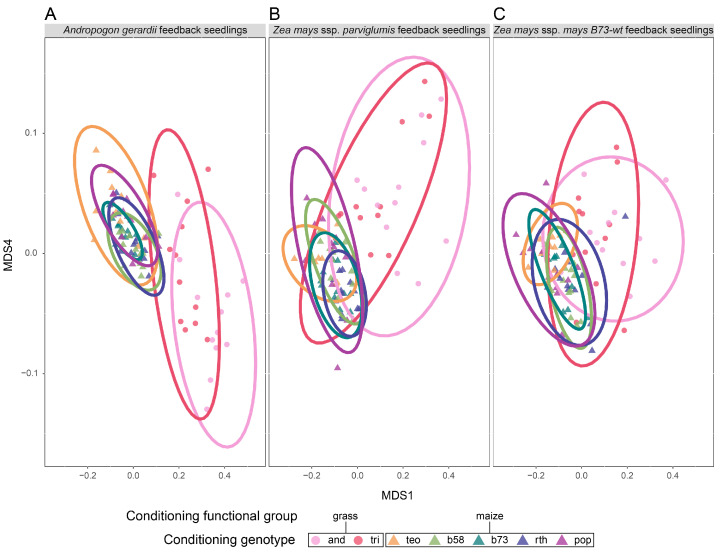
Effects of feedback genotype and conditioning genotype on the final soil nutrient composition. Non-metric multidimensional scaling (NMDS) analysis using Gower’s distance on scaled soil nutrient concentrations was the multivariate response variable parsed into (**A**) *Andropogon gerardii*, (**B**) *Zea mays* ssp. *parviglumis*, and (**C**) *Zea mays* ssp. *mays B73*-*wt* feedback seedlings. The composition of the soil nutrient concentrations included carbon and nitrogen percentages in soil, along with milligrams of phosphorus, potassium, calcium, and magnesium per kilogram of soil analyzed. Colors and symbols indicate the conditioning genotypes and functional groups, respectively. The prairie grass species (circles) are pink (*Andropogon gerardii*; ‘and’) and red (*Tripsacum dactyloides*; ‘tri’). Maize genotypes are triangles, and the *Z. mays* ssp. *parviglumis* are light orange (teosinte, ‘teo’), while the *Z. mays* ssp. *mays* genotypes are in cooler colors: *b58* is light green, *B73*-*wt* is dark green, *B73*-*rth3* (‘rth’) is dark blue, and *HP301* (‘pop’) is purple. Ellipses represent the 95% confidence ellipse based on the standard deviation around the centroid. Refer to Table S4 for the corresponding statistical analyses. The corresponding permutational analysis of variance (perMANOVA) and tests of dispersion can be found in Table 4.

The final soil nutrient composition was significantly influenced by the feedback genotype, conditioning effects, and their interactions (Table 4; Figure 6). While all factors were statistically significant, the effect of prairie grass species versus maize-conditioned soils (the conditioning functional group) explained the greatest amount of variation in the final soil nutrient concentrations (Table 4: *R*^2^ = 0.20). The conditioning genotype nested within the functional group had the second highest variation explained for soil nutrient concentrations (Table 4: *R*^2^ = 0.09), followed by the interaction effect of feedback genotype × conditioning genotype and the feedback genotype main effect (Table 4: *R*^2^ = 0.07 and 0.05, respectively). With variation across the soil conditioning genotypes within each plant feedback genotype, the sum of all final soil nutrient concentrations was strongly influenced by the conditioning functional groups.

The final soil phosphorus and potassium differed across conditioning genotypes for all feedback genotypes, while *A. gerardii* and maize *B73*-*wt* feedback plants also had differences between conditioning genotypes in final soil carbon and magnesium concentrations, while maize *B73*-*wt* feedback plants alone showed significant differences in the final soil nitrogen (Table 3; Figure S5). All but soil phosphorus and potassium concentrations varied by feedback genotype in unconditioned soils compared to plant-conditioned soils (Figure S5): teosinte feedback plants had greater differences in the final soil nutrient concentrations in self-conditioned soils than pre-conditioned or other plant-conditioned soils (Figure S5M,N,Q,R); maize *B73*-*wt* feedback soil nutrient concentrations were similar across soil conditioning genotypes, pre-conditioned, and unconditioned soils (Figure S5G,H,J,K); and *A. gerardii* feedback soil carbon and nitrogen concentrations were the lowest in unconditioned soils compared to pre-conditioned and plant-conditioned soils (Figure S5A,B), while soil calcium and magnesium concentrations were similar across soil treatments (Figure S5E,F). For soil carbon, final soils of *A. gerardii* feedback plants conditioned by maize *b58* soils showed marginally lower carbon concentrations compared to all but the self-conditioned soils (*p_adj_* ≤ 0.063), and the final soils of maize *B73*-*wt* feedback plants had lower carbon concentrations when conditioned by maize *b58* and *T. dactyloides* compared to teosinte, self-conditioned, and maize *HP301* soils (*p_adj_* ≤ 0.020) (Figure S5A,G). For soil nitrogen, the final soils of maize *B73*-*wt* feedback plants had higher nitrogen concentrations from soils conditioned by maize *HP301* than *T. dactyloides* (*p_adj_* = 0.043) (Figure S5H). Soil calcium concentration did not differ between conditioning genotypes for any feedback genotype (Table 3, Figure S5E,K,Q). For soil magnesium, final soils of *A. gerardii* feedback plants conditioned by maize b58 and *B73*-*wt* soils had lower magnesium concentrations than soils conditioned by *T. dactyloides*, teosinte, and maize *HP301*(*p_adj_* ≤ 0.034) (Figure S5F), while the final soils of maize *B73*-*wt* feedback plants conditioned by maize *b58* had lower magnesium concentrations than soils conditioned by prairie grass species and maize *HP301* (*p_adj_* ≤ 0.038) (Figure S5L). The final soils across the feedback genotypes in unconditioned soils had significantly higher phosphorus and potassium concentrations than plant-conditioned soils, while the pre-conditioned average was consistently the highest (Figure S5C,D,I,J,O,P). For the soil conditioning genotypes, the final soil phosphorus and potassium concentrations were significantly higher in soils conditioned by *A. gerardii* and *T. dactyloides* (prairie grass species) compared to maize genotypes (except soils of *B73*-*wt* feedback plants conditioned by *B73*-*rth3*) across the feedback genotypes (Table 3: *p_adj_* ≤ 0.029) (Figure S5C,D,I,J,O,P). Overall, the soil phosphorus and potassium were strongly influenced by the conditioning functional groups for all feedback genotypes, while the final soil carbon, nitrogen, and magnesium differed between individual conditioning genotypes in similar patterns across the feedback genotypes.

Differing from the final soil nutrient composition, the feedback foliar nutrient composition was more strongly influenced by the feedback genotype than the conditioning genotype or functional group (Table S4, Figure 5 and Figure S4). While all factors were statistically significant, the feedback genotype explained the greatest amount of variation in the foliar nutrient concentrations (Table S4: *R*^2^ = 0.30), and the interaction effect of the feedback genotype × conditioning genotype also explained the second largest amount (Table S4: *R*^2^ = 0.30). The conditioning functional groups had less variation explained for the final soil nutrient concentrations (Table S4: *R*^2^ = 0.12), and the smallest variation was explained by the effect of the conditioning genotype nested within the functional group (Table S4: *R*^2^ = 0.02). Foliar nutrient incorporation was strongly influenced by the feedback genotype, but still had significant responses to the conditioning functional groups.

## 4. Discussion

**Figure 7:**
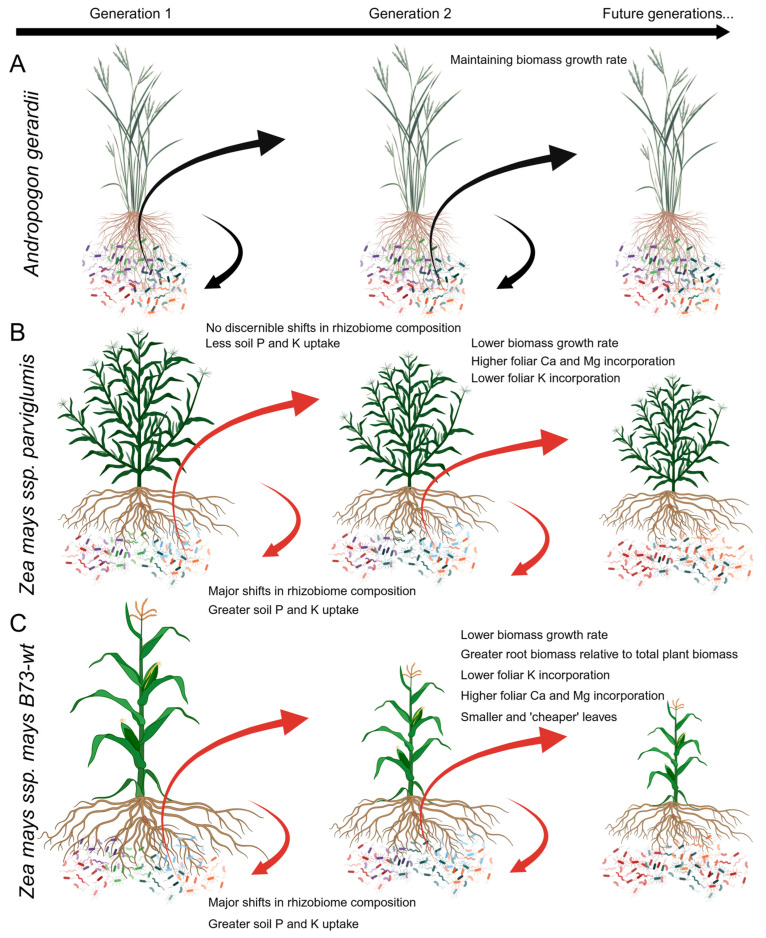
Plant soil feedback effects in monoculture. A synthesis of the conditioning and feedback effects for (**A**) *Andropogon gerardii*, (**B**) *Zea mays* ssp. *parviglumis*, and (**C**) *Zea mays* ssp. *mays B73*-*wt* feedback seedlings in self-conditioned soils—from the same conditioning genotype or conditioning functional group—relative to soils conditioned by ‘other’ genotypes, across generations of growth. Colors and arrows refer to negative (red), nonsignificant or zero (black), and positive (green) effects between plants and rhizobiomes (arrows leading from plants to microbes in the same column) and feedback effects on successive generations of plant growth in monoculture (arrows spanning across columns). The text refers to the specific processes identified in the results of this study for each feedback genotype. This figure was created using biorendr.com.

Soil health is considered a key component of sustainable and high-yielding agricultural systems [3,7,10,119]. Soil domestication erodes soil health [22,71,120], and our study of agricultural and wild species in the Poaceae showed that nutrient and microbiome-mediated PSF might be partly responsible for soil domestication in cereal crop systems. We found that PSF was stronger and more negative for maize than for wild prairie grass species in the same tribe (Andropogoneae) and that effects on both soil nutrient availability and rhizosphere microbial communities are likely causal factors (Figure 7). Specifically, soil conditioning with maize genotypes reduced the concentrations of most soil nutrients and significantly shifted the microbial community structure, resulting in slower growth rates as well as compensatory phenotypic changes, especially in maize *B73*-*wt* and teosinte feedback plants grown in maize-conditioned soil. These effects were also observed after conditioning by the ancestral maize species, teosinte, although not as strongly as with conditioning by the maize genotypes. Although it has been documented that maize cultivation reduces soil nutrient concentrations [120,121,122,123], our study is one of the first to show that negative PSF in agricultural contexts is also likely to be caused by shifts in the soil microbial community, although we cannot partition the relative contributions of these effects to the negative PSF caused by maize genotypes and teosinte. In contrast, soils conditioned by prairie grasses had generally beneficial effects on the growth and phenotypes of maize *B73*-*wt* and teosinte plants, likely owing to minimal reductions in most soil nutrients and only small shifts in the microbial community. Most prairie grasses have lower nutrient demands than maize [124], and, with the exceptions of calcium and magnesium, *A. gerardii* depleted soil nutrients less than maize conditioning genotypes. Since the pre-conditioned soil inoculum originated from a remnant natural prairie, it makes sense that conditioning with prairie grass would result in minimal shifts in the microbial community compared to conditioning by both maize genotypes and teosinte. Our study showed that soil nutrient and rhizobiome-mediated negative PSF involving maize could become evident even in a short amount of time, suggesting that such effects may arise soon after the conversion from prairie to agricultural fields or even between crop rotations.

The advantage of using a greenhouse pot experiment is the ability to isolate PSF by better controlling other environmental conditions affecting plant growth that could confound inferences in a field experiment. Moreover, given the rarity of intact prairies in the Great Plains of North America, it would have been challenging to conduct this experiment in the field and may have also had undesirable consequences for prairie diversity conservation. Although there are uncertainties in translating the results of our pot experiment to the field, we hypothesize that similar soil nutrient and microbe-mediated PSF processes are also operating to domesticate soils in agricultural systems where the same cereal crop is grown in monocultures over successive years. Our study suggests that these PSF processes are likely to have negative effects on cereal crop productivity, but further studies in the field are needed to evaluate this hypothesis.

### 4.1. Effects of Conditioning on Soil Microbial Communities

Conditioning by different plant genotypes and species caused variations in the soil microbial community composition. Based on presence–absence metrics, microbial communities in maize-conditioned soils differed from all other conditioned soils, as well as from the unconditioned controls and pre-conditioned soils. The microbial community of the preconditioned soil was determined by an inoculant from a tallgrass prairie that had no agricultural influences and was likely shaped by PSF operating at the site of soil collection, where *A. gerardii* was common. Thus, the strong effects of conditioning by maize genotypes on the soil microbiome were particularly evident in comparison with the minimal effects of conditioning by the prairie grasses, *A. gerardii* and *T. dactyloides*. The post-conditioning soil microbial ASV composition for plant conditioning genotypes was also strongly influenced by the absence of root hairs, as communities in soils conditioned by the root hairless mutant *Z. mays B73*-*rth3* differed significantly from those conditioned by several other maize genotypes as well as by both prairie grass species using both relative abundance-weighted and presence–absence metrics. This observation is consistent with a previous study [43] that found differences in the soil microbial ASV composition between *Z. mays B73*-*wt* and *rth3*. In abundance-weighted analyses, the effects of conditioning on the soil microbial community were not always statistically significant, demonstrating the importance of examining multiple measures of microbial community structure.

Genera in Verrucomicrobia (*Candidatus Xiphinematobacter* spp.) and a genus in the Burkholderiaceae family (*Massilia* spp.) were prevalent across all plant-conditioned rhizosphere communities, especially maize genotypes, in comparison to pre-conditioned and unconditioned soils. Maize-conditioned communities also shared *Sphingomonas* spp., along with unclassified genera in the Microscillaceae and Pedosphaeraceae. The relative abundance of Verrucomicrobia has been found to be higher in grasslands than in other ecosystems [125,126], and it was present in the preconditioned soils, aligning with the source of the inoculum microbial community. However, this study also showed Verrucomicrobia was strongly indicative of soils conditioned by maize *B73*-*rth3* rather than prairie grass species, suggesting the hypothesis of an understudied recruitment of Verrucomicrobia by the maize root system. In contrast, Actinobacteria genera were indicative of *A. gerardii*, including *Streptomyces* spp., which have been shown to produce several antibiotics that can promote pathogen defense in soil communities [127]. A future study could examine how long these conditioning effects by plant genotypes on the microbial community persist in field contexts.

### 4.2. Plant–Soil Feedback on Plant Growth, Phenotypic Traits, and Soil Nutrients

Maize feedback plants exhibited negative PSF resulting from conditioning by maize genotypes, as both maize *B73*-*wt* and teosinte grew faster in soils conditioned by wild prairie grass species compared to the more closely related *Zea mays* genotypes. The final soil nutrient concentrations in maize-conditioned soils were not significantly different (Ca) or were substantially lower (P, K) than in prairie grass-conditioned soils, which may partly explain the poorer performance of maize *B73*-*wt* and teosinte feedback plants in maize versus prairie grass-conditioned soils. This interpretation is supported by the fact that maize *B73*-*wt* allocated less mass to roots and grew larger leaves with higher nutrient (K) concentrations in prairie grass versus maize-conditioned soil, in conjunction with ‘cheaper’ leaf tissue (SLA) in maize-conditioned compared to maize-conditioned soils. The negative PSF of maize feedback plants could arise from a greater frequency of antagonistic microbial taxa in maize-conditioned soil, or from more beneficial microbial assemblages in prairie grass-conditioned soil. A future study could use metagenomic sequencing to examine the functional composition of microbial communities in maize versus prairie grass-conditioned soil to identify which mechanism is more important. Conversely, the performance and phenotypic variation of *A. gerardii* showed no significant variation among conditioning genotypes and little evidence of PSF.

In this study, the feedback phase was kept shorter than the conditioning phase so that PSF, if present, could be observed. Within the three months of conditioning, maize genotypes shifted the microbial community composition and soil nutrient concentrations compared to conditioning with wild prairie grass species and showed feedback responses after only one month of feedback. Considering that maize genotypes are often grown to yield in ca. 2–3 months and cultivated at high plant densities, our study suggests that negative PSF is likely to develop quickly in agricultural fields, although studies have sometimes shown that PSF is stronger in greenhouse experiments than in the field [71,128,129,130]; however, the ability to limit variations in other environmental conditions in the greenhouse may help make the PSF more visible. The reductions in soil nutrients and changes to the soil microbial community caused by PSF-mediated effects of repeated maize monoculture would require greater inputs of fertilizers to maintain crop productivity [22,122,131], but fertilizer would be unlikely to prevent domestication of the soil microbial community.

Soil domestication could be mitigated by leveraging PSF-mediated ecological complementarity effects [31,130,132] in agricultural systems. Wild prairie grasses or other cover crop and intercropping species that have lower nutrient demands than maize and that promote more beneficial soil microbiomes could slow effects of soil domestication [19,22,29]. However, whether PSFs are important in agricultural systems and the mecahnisms driving them have been largely uninvestigated [129]. Our study demonstrates that positive PSFs created through ecological complementarity could be an important tool for improving crop productivity. For example, many prairie grasses are perennials, so their nutrient uptake and use strategies differ from maize genotypes, which do not survive past a growing season and invest heavily in fast growth, maturation, and seed production. These differences in nutrient uptake and use were evident in our results: when soil nutrient concentrations were affected by conditioning, they were more affected by conditioning with maize genotypes than with prairie grasses, and foliar concentrations of most nutrients in maize and teosinte grown in prairie grass-conditioned soil were higher than in maize-conditioned soil, especially for K and P. In contrast, Ca and Mg were strongly depleted by grass conditioning, resulting in lower foliar concentrations of these nutrients in maize *B73*-*wt* and teosinte when grown in prairie grass-conditioned soil. A study on intercropping systems showed complementarity effects between plant families, such as nitrogen-fixing legumes versus grasses [7], but more studies are focusing on the differential effects on soil nutrients of domesticated species and their wild relatives in the same family [26,78], as in our study within the Poaceae.

The final soil nutrients showed residual effects of conditioning genotype even after the feedback plants grew in the conditioned soils, supporting the hypothesis that long-term PSF mediated by differences in nutrient uptake may be a driver of nutrient concentrations in plant communities. Shifts in soil nutrient concentrations were likely to be a product of the genotype-dependent nutrient uptake, litter input (likely senescence of fine roots), and root exudation by both feedback and conditioning plants, as well as by conditioning-mediated changes in the microbial community and their metabolic dynamics [29,69,133] that differentially affected organic matter decomposition and nutrient cycles. Evidence for the latter includes the fact that soil carbon, which is not a nutrient taken up by plants, varied significantly among conditioning genotypes and also increased relative to preconditioning and the negative control soils. Fluctuations in carbon are often driven by plant litter decomposition or exudation [134,135]. While this study did not define the exudate profiles of conditioning genotypes, many of the maize genotypes are known to vary [43,61]. Residual fine root tissues may have also remained in soils at the end of the experiment and could account for some of the differences in soil carbon. Soil microbial communities, including those associated with plant roots, both affect and are affected by soil nutrient availability [136,137,138,139], which, combined with nutrient uptake and litter production by plants, leads to complex interactions that are difficult to parse. Regardless, these interactions are likely to have led to compounded direct and indirect effects shaping the soil environment that the feedback plants experienced, producing significant PSF in maize *B73*-*wt* and teosinte.

Consistent with their lack of significant growth responses to soil conditioning, *A. gerardii* plants also did not exhibit strong conditioning effects on phenotypic traits. *A. gerardii* plants only had greater root length per unit of plant biomass in soils conditioned by prairie grasses, suggesting a greater investment in soil resource uptake capacity, perhaps in response to the depleted soil Ca and Mg. However, there were no other significant compensatory phenotypic changes in response to soil conditioning, and the differences in RLR did not extend to differences in the condition functional groups. These results suggest that *A. gerardii* growth and phenotypes may be relatively resilient to changes in the soil environment, at least compared to *Zea mays* genotypes. Another possible explanation is that since the seeds of *A. gerardii* used in our experiment were open-pollinated, genotypic variation within conditioning treatments obscured the ability to detect differences. However, the teosinte seeds were also open-pollinated, but teosinte exhibited significant conditioning-mediated effects on growth rate and foliar nutrient concentrations (K, Ca, and Mg), but no other phenotypic traits.

## 5. Conclusions

By integrating data on soil microbial communities, nutrients, plant growth, and phenotypes, this study contributes novel insights concerning the mechanisms causing PSF. Specifically, it identified plant-driven direct effects on soil nutrient availability and microbial communities that produced dramatically different soil legacy effects between wild prairie and domesticated crop species in the Poaceae. In terms of future prospects, by advancing the understanding of PSF in wild and domesticated grass species, this study’s findings can help improve the management of agroecosystems in several ways. By elucidating the effects of maize monoculture on soil health, this study’s findings can identify ways to leverage ecological interactions to improve sustainability in agriculture and soil ecosystems [77,140,141]. PSF may also be an important mechanism influencing post-agricultural succession, thereby informing future efforts to restore prairies from abandoned agricultural fields [28].

## Figures and Tables

**Table 1:** Plant growth rates, phenotypic traits, and final soil nutrient concentrations analyzed for *Andropogon gerardii*, *Zea mays* ssp. *parviglumis*, and *Zea mays* ssp. *mays B73* wild-type seedlings of the feedback phase of a plant–soil feedback experiment. Trait abbreviations, a brief description, and the corresponding units for each growth rate, phenotypic trait, leaf, and final soil nutrient concentrations analyzed in this study are provided. The phenotypic traits were analyzed separately for each feedback genotype at the level of the biological replicate (pot).

Abbreviation	Description	Units
***Plant performance***		
** Total bm gr**	total biomass growth rate per month	g (harvested plant biomass—initial plant biomass)/month
***Phenotypic traits***		
** LMR**	leaf mass ratio	g leaf dry weight/g plant biomass
** LAR**	leaf area ratio	cm^2^/g leaf dry weight
** SLA**	specific leaf area	cm^2^ leaf area/g leaf dry weight
** LDMC**	leaf dry matter content	g leaf dry weight/g leaf fresh weight
** LTD**	leaf tissue density	g leaf dry weight/cm^3^ leaf volume
** Leaf thick**	mean leaf thickness	mm
** LA**	mean area of a leaf	cm^2^
** RMR**	root mass ratio	g root biomass/g plant biomass
** RLR**	root length ratio	cm root length/g plant biomass
** SRL**	specific root length	cm root/g root dry weight
** RTD**	root tissue density	g total root dry weight/cm^3^ root volume
*Leaf nutrient concentrations*		
** Leaf C**	leaf carbon	Percent carbon
** Leaf N**	leaf nitrogen	Percent nitrogen
** Leaf P**	leaf phosphorus	Percent phosphorus
** Leaf K**	leaf potassium	Percent potassium
** Leaf Ca**	leaf calcium	Percent calcium
** Leaf Mg**	leaf magnesium	Percent magnesium
*Soil nutrient concentrations*		
** Soil C**	soil carbon	Percent carbon
** Soil N**	soil nitrogen	Percent nitrogen
** Soil P**	soil phosphorus	mg phosphorus/kg soil
** Soil K**	soil potassium	mg potassium/kg soil
** Soil Ca**	soil calcium	mg calcium/kg soil
** Soil Mg**	soil magnesium	mg magnesium/kg soil

**Table 2:** Effects of conditioning genotype on the microbial community composition and dispersion. Permutational multivariate analyses of variance (perMANOVA) and homogeneity of dispersion analyses were performed based on abundance-weighted (Bray-Curtis) and presence–absence (Jaccard) distance metrics to test the effects of conditioning genotype nested within prairie grass versus maize-conditioning groups (functional group) on the microbial community composition described by the microbial amplicon sequence variants (ASVs). The perMANOVA numerator degrees of freedom (*df*) were 1 for functional group and 5 for conditioning genotype nested within the functional group, with denominator degrees of 72. Tests of dispersion were conducted for the main effect of the functional group with each ordination method, where the numerator *df* was 1 for the functional group, with denominator degrees of 77. See Figure 3 for the corresponding ordination plots.

	perMANOVA			Test of Dispersion	
Factor	*F*	Probability (*p*)	R^2^	*F*	Probability (*p*)
***Abundance*** **-*weighted***					
** Functional group**	1.32	0.001	0.02	1.37	0.246
** Functional group: conditioning genotype**	1.10	0.001	0.07	-	-
***Presence*** **–*absence***					
** Functional group**	1.23	0.001	0.02	0.68	0.411
** Functional group: conditioning genotype**	1.12	0.001	0.07	-	-

**Table 3:** Effecst of the conditioning genotype on the plant performance, phenotypic traits, leaf nutrients, and final soil nutrient concentrations of *A. gerardii*, *Z. mays* ssp. *parviglumis* (teosinte), and *Z. mays* ssp. *mays B73* wild type. Results of the type III analysis of variance tests for the mixed models for each feedback plant species of the fixed effect of conditioning genotype. All probabilities are controlled for the false discovery rate (FDR) using the Benjamini–Hochberg method. See Section 2.3.2 in the Methods for details.

Response Variable	*Andropogon* *gerardii*	*Z. mays* ssp. *mays B73*-*wt*	*Z. mays* ssp. *parviglumis*
***Plant performance***			
** Total bm gr**	*F*_6,75_ = 3.04 *p_adj_* = 0.235	*F*_6,63_ = 9.88 *p_adj_* < 0.001	*F*_6,65_ = 16.9 *p_adj_* < 0.001
***Phenotypic traits***			
** LMR**	*F*_6,62_ = 0.48 *p_adj_* = 1.000	*F*_6,64_ = 0.07 *p_adj_* = 1.000	*F*_6,65_ = 0.55 *p_adj_* = 1.000
** SLA**	*F*_6,74_ = 0.54 *p_adj_* = 1.000	*F*_6,62_ = 4.21 *p_adj_* = 0.029	*F*_6,65_ = 2.59 *p_adj_* = 0.594
** LDMC**	*F*_6,64_ = 1.54 *p_adj_* = 1.000	*F*_6,63_ = 0.82 *p_adj_* = 1.000	*F*_6,74_ = 0.93 *p_adj_* = 1.000
** LTD**	*F*_6,74_ = 0.53 *p_adj_* = 1.000	*F*_6,64_ = 0.58 *p_adj_* = 1.000	*F*_6,72_ = 0.52 *p_adj_* = 1.000
** LAR**	*F*_6,74_ = 0.61 *p_adj_* = 1.000	*F*_6,63_ = 1.21 *p_adj_* = 1.000	*F*_6,65_ = 2.21 *p_adj_* = 1.000
** LA**	*F*_6,63_ = 1.70 *p_adj_* = 1.000	*F*_6,61_ = 21.85 *p_adj_* < 0.001	*F*_6,65_ = 3.22 *p_adj_* = 0.182
** RMR**	*F*_6,62_ = 1.03 *p_adj_* = 1.000	*F*_6,63_ = 8.80 *p_adj_* < 0.001	*F*_6,66_ = 0.88 *p_adj_* = 1.000
** RLR**	*F*_6,63_ = 3.96 *p_adj_* = 0.046	*F*_6,74_ = 1.21 *p_adj_* = 1.000	*F*_6,65_ = 1.69 *p_adj_* = 1.000
** SRL**	*F*_6,58_ = 3.11 *p_adj_* = 0.239	*F*_6,62_ = 1.74 *p_adj_* = 1.000	*F*_6,53_ = 1.71 *p_adj_* = 1.000
** RTD**	*F*_6,74_ = 3.00 *p_adj_* = 0.257	*F*_6,63_ = 0.64 *p_adj_* = 1.000	*F*_6,64_ = 0.97 *p_adj_* = 1.000
***Leaf nutrient concentrations***			
** Leaf C**	*F*_6,70_ = 0.57 *p_adj_* = 1.000	*F*_6,74_ = 1.72 *p_adj_* = 1.000	*F*_6,74_ = 3.10 *p_adj_* = 0.211
** Leaf N**	*F*_6,74_ = 2.05 *p_adj_* = 1.000	*F*_6,63_ = 4.22 *p_adj_* = 0.029	*F*_6,64_ = 2.67 *p_adj_* = 0.516
** Leaf P**	*F*_6,60_ = 1.21 *p_adj_* = 0.315	*F*_6,63_ = 0.70 *p_adj_* = 1.000	*F*_6,64_ = 1.58 *p_adj_* = 1.000
** Leaf K**	*F*_6,70_ = 1.96 *p_adj_* = 0.083	*F*_6,63_ = 54.68 *p_adj_* < 0.001	*F*_6,64_ = 22.17 *p_adj_* < 0.001
** Leaf Ca**	*F*_6,57_ = 2.80 *p_adj_* = 0.110	*F*_6,63_ = 14.46 *p_adj_* < 0.001	*F*_6,74_ = 8.90 *p_adj_* < 0.001
** Leaf Mg**	*F*_6,61_ = 0.91 *p_adj_* = 0.493	*F*_6,63_ = 28.94 *p_adj_* < 0.001	*F*_6,74_ = 13.05 *p_adj_* < 0.001
***Soil nutrient concentrations***			
** Soil C**	*F*_6,60_ = 3.52 *p_adj_* = 0.036	*F*_6,61_ = 4.62 *p_adj_* = 0.005	*F*_6,60_ = 2.61 *p_adj_* = 0.194
** Soil N**	*F*_6,60_ = 2.43 *p_adj_* = 0.165	*F*_6,61_ = 3.49 *p_adj_* = 0.023	*F*_6,61_ = 1.59 *p_adj_* = 0.630
** Soil P**	*F*_6,60_ = 11.43 *p_adj_* < 0.001	*F*_6,51_ = 18.88 *p_adj_* < 0.001	*F*_6,63_ = 25.24 *p_adj_* < 0.001
** Soil K**	*F*_6,70_ = 120.38 *p_adj_* < 0.001	*F*_6,74_ = 24.50 *p_adj_* < 0.001	*F*_6,72_ = 46.59 *p_adj_* < 0.001
** Soil Ca**	*F*_6,70_ = 1.69 *p_adj_* = 0.521	*F*_6,50_ = 1.09 *p_adj_* = 0.417	*F*_6,61_ = 2.42 *p_adj_* = 0.194
** Soil Mg**	*F*_6,70_ = 3.12 *p_adj_* = 0.053	*F*_6,74_ = 3.14 *p_adj_* = 0.015	*F*_6,61_ = 2.34 *p_adj_* = 0.194

**Table 4:** Effects of feedback genotype, conditioning genotype, and their interactions on the final soil nutrient concentrations. Permutational multivariate analyses of variance (perMANOVA) and homogeneity of dispersion analyses were performed based on an abundance-weighted (Gower) distance metric to separately test the effects of the feedback genotype, conditioning genotype, their interaction, and the conditioning functional group on the final soil nutrient composition. The perMANOVA numerator degrees of freedom (*df*) were 1 for functional group, 2 for feedback genotype, 5 for conditioning genotype nested within their respective functional group, and 12 for the interaction of conditioning and feedback genotypes, with denominator degrees of 223. Separate tests of dispersion were conducted for the main effects of feedback genotype and functional group, where the numerator *df* were 2 for feedback genotype and 1 for functional group, with denominator degrees of 241 and 242, respectively. The homogeneity of multivariate dispersion was assessed by calculating the ordination to estimate the distances from the centroid, followed by an ANOVA for separate analyses of the conditioning functional group.

	perMANOVA			Test of Dispersion	
Factor	*F*	Probability (*p*)	R^2^	*F*	Probability (*p*)
Functional group	75.56	0.001	0.20	30.36	<0.001
Feedback genotype	9.93	0.001	0.05	0.51	0.599
Functional group: conditioning genotype	6.55	0.001	0.09	-	-
Feedback × conditioning genotype	2.26	0.001	0.07	-	-

## Data Availability

Raw 16S rRNA gene amplicon sequences can be found in the NCBI SRA (PRJNA1049996). The example R code for analyses is available in Appendix S3. Plant phenotypic traits, including leaf and soil nutrient concentrations, microbial qPCR copy numbers, and the microbial metadata file used for analysis can be found in the Dryad data repository (https://doi.org/10.5061/dryad.0p2ngf26x).

## Supplementary Materials

The following supporting information can be downloaded at: https://www.mdpi.com/article/10.3390/microorganisms11122978/s1, Table S1: Numbers of reads (amplicons) and ASVs after filtering across conditioning genotypes in the rhizosphere; Table S2: Effects of conditioning genotype on soil microbial abundance and community alpha diversity metrics; Table S3: Microbial indicator taxa of conditioning genotype rhizobiomes; Table S4: Effects of feedback genotype, conditioning genotype, and their interaction on foliar nutrient composition; Figure S1: Variation in taxonomic classification for pre-conditioned, unconditioned controls, and different conditioning genotype soil microbial communities; Figure S2: Variation in ASV microbial community composition between soil conditioning types; Figure S3: Shared and unique taxa for maize and Poaceae rhizobiomes; Figure S4: Variation in foliar nutrient concentrations of feedback plants among conditioning genotypes and unconditioned controls; Figure S5: Variation in final soil nutrient concentrations in pots of feedback plants among conditioning genotypes, pre-conditioned soils, and the unconditioned controls.
