# Supplementary material for: Nutrient and Microbiome-Mediated Plant–Soil Feedback in Domesticated and Wild Andropogoneae: Implications for Agroecosystems
Source: Microorganisms. 2023 Dec 13;11(12):2978. doi: 10.3390/microorganisms11122978 (PMC10745641; doi:10.3390/microorganisms11122978)
Supplement: Supplementary file 1 [file microorganisms-11-02978-s001.zip › microorganisms-2670377-supplementary.pdf]

Nutrient and Microbiome-Mediated Plant–Soil Feedback in Domesticated and Wild Andropogoneae: Implications for Agroecosystems

Amanda Quattrone [1,2,3,*], Yuguo Yang [2], Pooja Yadav [2], Karrie A. Weber [2,4,5] and Sabrina E. Russo [2,3,*]

[1] Complex Biosystems Ph.D. Program, University of Nebraska-Lincoln, Lincoln, NE 68583-0851, USA
[2] School of Biological Sciences, University of Nebraska-Lincoln, Lincoln, NE 68588-0118, USA; yuguo@huskers.unl.edu (Y.Y.)
[3] Center for Plant Science Innovation, University of Nebraska-Lincoln, Lincoln, NE 68583-0705, USA
[4] Department of Earth and Atmospheric Sciences, University of Nebraska-Lincoln, Lincoln, NE 68588-0340, USA
[5] Daugherty Water for Food Institute, University of Nebraska, Lincoln, NE 68588-6203, USA
* Correspondence: amanda.quattrone@huskers.unl.edu (A.Q.); srusso2@unl.edu (S.E.R.)

Appendix S1: Supplemental Methods

Conditioning phase: Seed collection

Seeds of *Zea mays* ssp. *mays B73-wt*, *B73-rth3*, *b58*, and *HP301* were obtained from in-house collections bulked in the greenhouse through self-pollination (Lincoln, Nebraska). For the *B73-rth3* seedlings that were used to generate seeds, the absence of both *rth3* alleles was confirmed through PCR. Seed batches of these seedlings were tested for the root-hairless phenotype by germinating 10 seeds per ear on petri dishes to a primary root length of 10 cm and confirming the absence of root hair using a stereo microscope. *Zea mays ssp. parviglumis* seeds were bulked in field growth through open pollination by Petcher Seeds, LLC (Fruitdale, AL). Seeds of *Andropogon gerardii* were obtained from Prairie Legacy, LLC (Witt's End Homestead, NE). from locally sourced plants that were grown in species-specific gardens and open-pollinated. Seeds of *Tripsacum dactyloides* were collected from a prairie in southeastern Nebraska.

Appendix S2: Supplemental tables and figures

Table S1. Numbers of reads (amplicons) and ASVs after filtering across conditioning genotypes in the rhizosphere. Read counts and the number of amplicon sequence variants (ASVs) used in analyses. Negative control (unconditioned) soils were soils from pots with no plants that were treated identically to pots with plants. Preconditioned soils pertain to one biological replicate of the initial soil community prior to planting in pots.

	Abbreviation	Number of reads	Number of ASVs
**Maize functional group**		**155544**	**2878**
Zea mays parviglumis	teo	23523	588
Zea mays mays b58	b58	33599	827
Zea mays mays B73-wt	b73	23916	585
Zea mays mays B73-rth3	rth	39730	884
Zea mays mays HP301	pop	34776	833
**Prairie grass functional group**		**55469**	**1075**
Tripsacum dactyloides	tri	28800	649
Andropogon gerardii	and	26669	567
**Other soils**		NA	NA
Preconditioned soil	ini	2726	269
Unconditioned soils	neg	5862	177

Table S2. Effects of conditioning genotype on soil microbial abundance and community alpha diversity metrics. Type III analysis of variance (ANOVA) tests assessed the significance of the conditioning genotype effect using separate models with or without unconditioned soil controls from the conditioning phase. Tests were based on amplicon sequence variant (ASV) relative abundance. The observed richness was the total number of ASVs, diversity was measured using Shannon's diversity index and Fisher's alpha diversity index, and Simpson's evenness.

Response variable	With soil controls	No soil controls
**Log(abundance)**	$F_{7,69} = 0.84$<br>$p = 0.524$	$F_{6,65} = 0.87$<br>$p = 0.499$
**Observed ASV richness**	$F_{7,74} = 1.70$<br>$p = 0.121$	$F_{6,72} = 1.88$<br>$p = 0.097$
**Shannon's diversity**	$F_{7,74} = 1.46$<br>$p = 0.193$	$F_{6,72} = 1.66$<br>$p = 0.143$
**Fisher's alpha diversity**	$F_{7,74} = 1.71$<br>$p = 0.101$	$F_{6,72} = 2.01$<br>$p = 0.076$
**Simpson's evenness**	$F_{7,74} = 1.93$<br>$p = 0.077$	$F_{6,72} = 2.19$<br>$p = 0.053$

Table S3. Microbial indicator taxa of conditioning genotype rhizobiomes. A point biserial correlation analysis of rhizosphere amplicon sequence variants (ASVs) to assess for microbial taxa which are more prevalent in soils of specific conditioning genotypes or shared by conditioning genotypes. Indicator ASVs included a biserial correlation coefficient (r_{pb}) and correlation probability (p_{corr}) for significant ($p_{corr} < 0.05$) comparisons within an associated conditioning genotype(s). Each ASV shows the phylum, family, and genus-level classification trained using the SILVA v 132 database. Unclassified taxonomic levels from the SILVA database were marked with "NA".

Conditioning genotype(s)	r_{pb}	p_{corr}	Phylum	Family	Genus
and	0.50	0.002	*Actinobacteria*	*Nocardioidaceae*	*Kribbella*
and	0.47	0.001	*Actinobacteria*	*Micromonosporaceae*	*Actinocatenispora*
and	0.45	0.008	*Actinobacteria*	*Micromonosporaceae*	*Actinocatenispora*
and	0.42	0.029	*Actinobacteria*	*Streptomycetaceae*	*Streptomyces*
and	0.42	0.032	*Actinobacteria*	*Streptomycetaceae*	*Streptomyces*
and	0.42	0.030	*Actinobacteria*	*Nocardioidaceae*	*Marmoricola*
and	0.42	0.031	*Patescibacteria*	*NA*	*NA*
and	0.41	0.018	*Proteobacteria*	*Xanthomonadaceae*	*Luteimonas*
and	0.41	0.029	*Proteobacteria*	*Rhodanobacteraceae*	*Dyella*
and	0.41	0.036	*Patescibacteria*	*NA*	*NA*
and	0.41	0.036	*Proteobacteria*	*Rhodanobacteraceae*	*Fulvimonas*
and	0.41	0.024	*Patescibacteria*	*Saccharimonadaceae*	*NA*
and	0.40	0.025	*Proteobacteria*	*Rhodanobacteraceae*	*Fulvimonas*
and	0.40	0.032	*Bacteroidetes*	*Chitinophagaceae*	*NA*
and	0.39	0.031	*Actinobacteria*	*Micromonosporaceae*	*Actinocatenispora*
and	0.37	0.031	*Bacteroidetes*	*Chitinophagaceae*	*Niastella*
and	0.36	0.030	*Actinobacteria*	*Streptomycetaceae*	*Streptomyces*
and	0.36	0.048	*Actinobacteria*	*Streptomycetaceae*	*Allostreptomyces*
and	0.35	0.039	*Actinobacteria*	*67-14*	*NA*
and	0.32	0.030	*Actinobacteria*	*Streptomycetaceae*	*Streptomyces*
b58	0.42	0.018	*Bacteroidetes*	*Microscillaceae*	*NA*
b58	0.38	0.026	*Proteobacteria*	*Burkholderiaceae*	*Ralstonia*
b73	0.57	0.001	*Actinobacteria*	*Intrasporangiaceae*	*Janibacter*
b73	0.40	0.047	*Proteobacteria*	*Burkholderiaceae*	*Burkholderia-Caballeronia-Paraburkholderia*
b73	0.39	0.039	*Planctomycetes*	*Pirellulaceae*	*NA*
b73	0.37	0.044	*Verrucomicrobia*	*Pedosphaeraceae*	*NA*
b73	0.36	0.045	*Bacteroidetes*	*env.OPS 17*	*NA*
b73	0.34	0.048	*Proteobacteria*	*KD3-10*	*NA*
b73	0.34	0.048	*Proteobacteria*	*Sphingomonadaceae*	*Sphingomonas*
pop	0.51	0.003	*Actinobacteria*	*Intrasporangiaceae*	*Janibacter*
pop	0.45	0.014	*Proteobacteria*	*Burkholderiaceae*	*Pseudoduganella*
pop	0.41	0.018	*Proteobacteria*	*Burkholderiaceae*	*Ralstonia*

pop	0.40	0.028	*Gemmatimonadetes*	*Gemmatimonadaceae*	*Gemmatimonas*
pop	0.38	0.029	*Actinobacteria*	*Nocardioidaceae*	*Nocardioides*
rth	0.60	0.001	*Verrucomicrobia*	*Xiphinematobacteraceae*	*Candidatus Xiphinematobacter*
rth	0.46	0.004	*Verrucomicrobia*	*Xiphinematobacteraceae*	*Candidatus Xiphinematobacter*
rth	0.44	0.015	*Verrucomicrobia*	*Pedosphaeraceae*	*NA*
rth	0.42	0.014	*Verrucomicrobia*	*Xiphinematobacteraceae*	*Candidatus Xiphinematobacter*
rth	0.41	0.013	*Gemmatimonadetes*	*Gemmatimonadaceae*	*Gemmatimonas*
rth	0.41	0.010	*Verrucomicrobia*	*Xiphinematobacteraceae*	*Candidatus Xiphinematobacter*
rth	0.40	0.013	*Verrucomicrobia*	*Xiphinematobacteraceae*	*Candidatus Xiphinematobacter*
rth	0.38	0.023	*Verrucomicrobia*	*Xiphinematobacteraceae*	*Candidatus Xiphinematobacter*
rth	0.37	0.049	*Verrucomicrobia*	*Pedosphaeraceae*	*NA*
teo	0.43	0.010	*Actinobacteria*	*67-14*	*NA*
teo	0.42	0.038	*Bacteroidetes*	*NA*	*NA*
teo	0.42	0.035	*Proteobacteria*	*Acetobacteraceae*	*Rhodovastum*
teo	0.41	0.034	*Gemmatimonadetes*	*Gemmatimonadaceae*	*Gemmatimonas*
teo	0.40	0.039	*Actinobacteria*	*Nocardioidaceae*	*Nocardioides*
teo	0.40	0.037	*Acidobacteria*	*NA*	*NA*
teo	0.39	0.029	*Chloroflexi*	*NA*	*NA*
teo	0.38	0.028	*Acidobacteria*	*Unknown_Family*	*Vicinamibacter*
teo	0.38	0.030	*Proteobacteria*	*Micropepsaceae*	*NA*
teo	0.38	0.030	*Planctomycetes*	*Phycisphaeraceae*	*NA*
teo	0.38	0.029	*Fibrobacteres*	*Fibrobacteraceae*	*Possible genus 04*
teo	0.37	0.027	*Actinobacteria*	*NA*	*NA*
teo	0.37	0.038	*Verrucomicrobia*	*Verrucomicrobiaceae*	*Prosthecobacter*
teo	0.36	0.028	*Verrucomicrobia*	*Pedosphaeraceae*	*NA*
tri	0.44	0.011	*Proteobacteria*	*Sphingomonadaceae*	*Sphingomonas*
tri	0.44	0.017	*Patescibacteria*	*Saccharimonadaceae*	*NA*
tri	0.43	0.018	*Actinobacteria*	*Streptomycetaceae*	*Streptomyces*
tri	0.41	0.020	*Actinobacteria*	*Streptomycetaceae*	*Streptomyces*
tri	0.41	0.020	*Proteobacteria*	*Dongiaceae*	*Dongia*
and:tri	0.36	0.047	*Bacteroidetes*	*env.OPS 17*	*NA*
b58:b73	0.45	0.010	*Verrucomicrobia*	*Opitutaceae*	*Opitutus*
b58:tri	0.36	0.041	*Proteobacteria*	*Burkholderiaceae*	*Massilia*
rth:teo	0.39	0.035	*Proteobacteria*	*Haliangiaceae*	*Haliangium*
and:b73: pop:rth	0.38	0.031	*Verrucomicrobia*	*Xiphinematobacteraceae*	*Candidatus Xiphinematobacter*

Table S4. Effects of feedback genotype, conditioning genotype, and their interaction on foliar nutrient composition. Permutational multivariate analyses of variance (perMANOVA) and homogeneity of dispersion analyses were performed based on an abundance-weighted (Gower) distance metric to separately test the effects of feedback genotype, conditioning genotype, their interaction, and conditioning functional group on the foliar nutrient composition. The perMANOVA numerator degrees of freedom (*df*) were 1 for functional group, 2 for feedback genotype, 5 for conditioning genotype nested within their respective functional group, and 12 *df* for the interaction of conditioning and feedback genotypes, with denominator degrees of 219. Separate tests of dispersion were conducted for main effects of feedback genotype and functional group, where the numerator *df* were 2 for feedback genotype, and 1 for functional group, with denominator degrees of 241 and 242, respectively. Homogeneity of multivariate dispersion was assessed by calculating the both the ordination methods to estimate the distances from the centroid, followed by an ANOVA for separate analyses of conditioning functional group.

	perMANOVA			Test of Dispersion	
**Factor**	***F***	**Probability**	R^2	***F***	**Probability**
**Functional group**	56.13	$p = 0.001$	0.12	30.36	$p < 0.001$
**Feedback genotype**	70.22	$p = 0.001$	0.30	0.51	$p = 0.599$
**Functional group: conditioning genotype**	2.30	$p = 0.001$	0.02	-	-
**Feedback × conditioning genotype**	3.99	$p = 0.001$	0.10	-	-

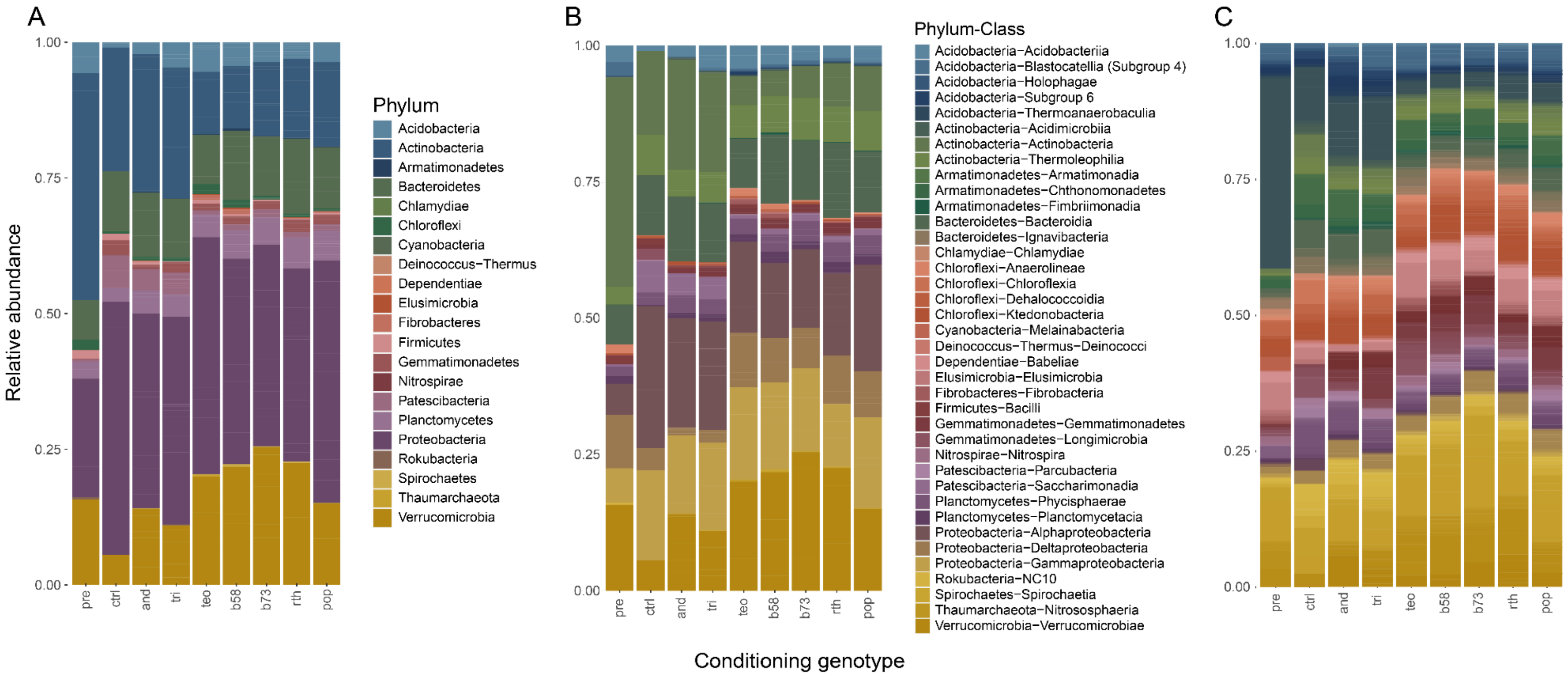

Figure S1. Variation in taxonomic classification for pre-conditioned, unconditioned controls, and different conditioning genotype soil microbial communities. Taxonomic bar plots of the relative abundance of amplicon sequence variants (ASVs) at the A) phylum, B) class level, grouped at the phylum level, and C) the genus level, grouped at the family level, nested in the class level of taxonomic classification (legend below). 47% of the genus-level classification was unclassified, while 15% of the family-level classification was unclassified by the SILVA v 132 database. 21 Phyla, 38 classes, 144 families, and 204 genera (315 unique genera, if "unclassified" genera are nested on a phylogenetic basis into family and class level groupings).

Class-Family-Genus

Acidimicrobiia-Iamiaceae-Iamia
Acidimicrobiia-Ilumatobacteraceae-Ilumatobacter
Acidimicrobiia-Ilumatobacteraceae-Unclassified
Acidimicrobiia-Unclassified-Unclassified
Acidobacteriia-Acidobacteriaceae_(Subgroup 1)-Acidipila
Acidobacteriia-Acidobacteriaceae_(Subgroup 1)-Edaphobacter
Acidobacteriia-Acidobacteriaceae_(Subgroup 1)-Occallatibacter
Acidobacteriia-Acidobacteriaceae_(Subgroup 1)-Terriglobus
Acidobacteriia-Koribacteraceae-Candidatus Koribacter
Acidobacteriia-Solibacteraceae_(Subgroup 3)-Bryobacter
Acidobacteriia-Solibacteraceae_(Subgroup 3)-Candidatus Solibacter
Acidobacteriia-Unclassified-Unclassified
Actinobacteria-Acidothermaceae-Acidothermus
Actinobacteria-Catenulisporaceae-Catenulispora
Actinobacteria-Cellulomonadaceae-Cellulomonas
Actinobacteria-Geodermatophilaceae-Blastococcus
Actinobacteria-Intrasporangiaceae-Janibacter
Actinobacteria-Intrasporangiaceae-Terrabacter
Actinobacteria-Microbacteriaceae-Lysinimonas
Actinobacteria-Microbacteriaceae-Microbacterium
Actinobacteria-Microbacteriaceae-Unclassified
Actinobacteria-Micrococcaceae-Paenarthrobacter
Actinobacteria-Micrococcaceae-Pseudarthrobacter
Actinobacteria-Micromonosporaceae-Actinocatenispora
Actinobacteria-Micromonosporaceae-Dactylosporangium
Actinobacteria-Micromonosporaceae-Unclassified
Actinobacteria-Mycobacteriaceae-Mycobacterium
Actinobacteria-Nocardiaceae-Nocardia
Actinobacteria-Nocardiaceae-Unclassified
Actinobacteria-Nocardioidaceae-Kribbella
Actinobacteria-Nocardioidaceae-Marmoricola
Actinobacteria-Nocardioidaceae-Nocardioides
Actinobacteria-Pseudonocardiaceae-Actinophytocola
Actinobacteria-Pseudonocardiaceae-Amycolatopsis
Actinobacteria-Pseudonocardiaceae-Pseudonocardia
Actinobacteria-Pseudonocardiaceae-Unclassified
Actinobacteria-Streptomycetaceae-Allostreptomyces
Actinobacteria-Streptomycetaceae-Kitasatospora
Actinobacteria-Streptomycetaceae-Streptomyces
Actinobacteria-Streptomycetaceae-Unclassified
Actinobacteria-Streptosporangiaceae-Nonomuraea
Actinobacteria-Thermomonosporaceae-Actinoallomurus
Actinobacteria-Thermomonosporaceae-Actinomadura
Actinobacteria-Thermomonosporaceae-Spirillospora
Actinobacteria-Unclassified-Unclassified
Alphaproteobacteria-Acetobacteraceae-Acidicaldus
Alphaproteobacteria-Acetobacteraceae-Rhodopila
Alphaproteobacteria-Acetobacteraceae-Rhodovastum
Alphaproteobacteria-Acetobacteraceae-Unclassified
Alphaproteobacteria-bac2nit3-Unclassified
Alphaproteobacteria-Beijerinckiaceae-Methylobacterium
Alphaproteobacteria-Caulobacteraceae-Asticcacaulis
Alphaproteobacteria-Caulobacteraceae-Caulobacter
Alphaproteobacteria-Caulobacteraceae-Phenylobacterium
Alphaproteobacteria-Caulobacteraceae-Unclassified
Alphaproteobacteria-Devosiaceae-Arsenicitalea
Alphaproteobacteria-Devosiaceae-Devosia
Alphaproteobacteria-Devosiaceae-Unclassified
Alphaproteobacteria-Dongiaceae-Dongia
Alphaproteobacteria-Elsteraceae-Elstera
Alphaproteobacteria-Elsteraceae-Unclassified
Alphaproteobacteria-Hyphomicrobiaceae-Hyphomicrobium
Alphaproteobacteria-Hyphomonadaceae-Hirschia
Alphaproteobacteria-Hyphomonadaceae-SWB02
Alphaproteobacteria-Inquilinaceae-Inquilinus
Alphaproteobacteria-Kaistiaceae-Kaistia
Alphaproteobacteria-KF-JG30-B3-Unclassified
Alphaproteobacteria-Magnetospiraceae-Unclassified
Alphaproteobacteria-Magnetospirillaceae-Phaeospirillum
Alphaproteobacteria-Magnetospirillaceae-Unclassified
Alphaproteobacteria-Micavibrionaceae-Micavibrio
Alphaproteobacteria-Micavibrionaceae-Unclassified
Alphaproteobacteria-Micropepsaceae-Micropepsis
Alphaproteobacteria-Micropepsaceae-Unclassified
Alphaproteobacteria-Midichloriaceae-Candidatus Jidaibacter
Alphaproteobacteria-Mitochondria-Unclassified
Alphaproteobacteria-Paracaedibacteraceae-Candidatus Paracaedibacter
Alphaproteobacteria-Reyranellaceae-Reyranella
Alphaproteobacteria-Rhizobiaceae-Allorhizobium-Neorhizobium-Pararhizobium-Rhizobium
Alphaproteobacteria-Rhizobiaceae-Mesorhizobium
Alphaproteobacteria-Rhizobiaceae-Shinella
Alphaproteobacteria-Rhizobiaceae-Unclassified
Alphaproteobacteria-Rhizobiales Incertae Sedis-Nordella
Alphaproteobacteria-Rhizobiales Incertae Sedis-Phreatobacter
Alphaproteobacteria-Rhodospirillaceae-Unclassified
Alphaproteobacteria-Rickettsiaceae-Unclassified
Alphaproteobacteria-SM2D12-Unclassified
Alphaproteobacteria-Sphingomonadaceae-Altererythrobacter
Alphaproteobacteria-Sphingomonadaceae-Ellin6055
Alphaproteobacteria-Sphingomonadaceae-Novosphingobium
Alphaproteobacteria-Sphingomonadaceae-Porphyrobacter
Alphaproteobacteria-Sphingomonadaceae-Qipengyuania
Alphaproteobacteria-Sphingomonadaceae-Sphingoaurantiacus
Alphaproteobacteria-Sphingomonadaceae-Sphingobium
Alphaproteobacteria-Sphingomonadaceae-Sphingomonas
Alphaproteobacteria-Sphingomonadaceae-Stakelama
Alphaproteobacteria-Sphingomonadaceae-Unclassified
Alphaproteobacteria-Unclassified-Unclassified
Alphaproteobacteria-URHD0088-Unclassified
Alphaproteobacteria-Xanthobacteraceae-Bradyrhizobium
Alphaproteobacteria-Xanthobacteraceae-Nitrobacter
Alphaproteobacteria-Xanthobacteraceae-Pseudolabrys
Alphaproteobacteria-Xanthobacteraceae-Unclassified
Anaerolineae-A4b-Unclassified
Anaerolineae-Anaerolineaceae-Unclassified
Anaerolineae-Unclassified-Unclassified
Armatimonadia-Unclassified-Unclassified
Babeliae-Unclassified-Unclassified
Bacilli-Alicyclobacillaceae-Tumebacillus
Bacilli-Bacillaceae-Bacillus
Bacilli-Bacillaceae-Unclassified
Bacilli-Paenibacillaceae-Paenibacillus
Bacilli-Paenibacillaceae-Unclassified
Bacilli-Planococcaceae-Chungangia
Bacilli-Planococcaceae-Unclassified
Bacilli-Unclassified-Unclassified
Bacteroidia-37-13-Unclassified
Bacteroidia-AKYH767-Unclassified
Bacteroidia-Chitinophagaceae-Chitinophaga
Bacteroidia-Chitinophagaceae-Ferruginibacter
Bacteroidia-Chitinophagaceae-Filimonas
Bacteroidia-Chitinophagaceae-Flaviaesturariibacter
Bacteroidia-Chitinophagaceae-Flavisolibacter
Bacteroidia-Chitinophagaceae-Flavitalea
Bacteroidia-Chitinophagaceae-Helimonas
Bacteroidia-Chitinophagaceae-Niastella
Bacteroidia-Chitinophagaceae-Niveitalea
Bacteroidia-Chitinophagaceae-Parafilimonas
Bacteroidia-Chitinophagaceae-Sediminibacterium
Bacteroidia-Chitinophagaceae-Segetibacter
Bacteroidia-Chitinophagaceae-Taibaiella
Bacteroidia-Chitinophagaceae-Terrimonas
Bacteroidia-Chitinophagaceae-Unclassified
Bacteroidia-Crocinitomicaceae-Fluviicola
Bacteroidia-Cytophagaceae-Cytophaga
Bacteroidia-Cytophagaceae-Sporocytophaga
Bacteroidia-env.OPS 17-Unclassified
Bacteroidia-Flavobacteriaceae-Flavobacterium
Bacteroidia-Microscillaceae-Chryseolinea
Bacteroidia-Microscillaceae-Ohtaekwangia
Bacteroidia-Microscillaceae-OLB12
Bacteroidia-Microscillaceae-Unclassified
Bacteroidia-NS11-12 marine group-Unclassified
Bacteroidia-Saprospiraceae-Unclassified
Bacteroidia-Sphingobacteriaceae-Arcticibacter
Bacteroidia-Sphingobacteriaceae-Mucilaginibacter
Bacteroidia-Sphingobacteriaceae-Pedobacter
Bacteroidia-Spirosomaceae-Dyadobacter
Bacteroidia-Unclassified-Unclassified
Bacteroidia-Weeksellaceae-Chryseobacterium
Blastocatellia_(Subgroup 4)-Blastocatellaceae-JGI 0001001-H03
Blastocatellia_(Subgroup 4)-Blastocatellaceae-Unclassified
Blastocatellia_(Subgroup 4)-Pyrinomonadaceae-RB41
Blastocatellia_(Subgroup 4)-Unclassified-Unclassified
Chlamydiae-cvE6-Unclassified
Chlamydiae-Simkaniaceae-Unclassified
Chloroflexia-JG30-KF-CM45-Unclassified
Chloroflexia-Roseiflexaceae-Unclassified
Chloroflexia-Thermomicrobiaceae-Nitrolancea
Chthonomonadetes-Chthonomonadaceae-Chthonomonas
Chthonomonadetes-Unclassified-Unclassified
Dehalococcoidia-Unclassified-Unclassified
Deinococci-Deinococcaceae-Deinococcus
Deltaproteobacteria-0319-6G20-Unclassified
Deltaproteobacteria-Archangiaceae-Anaeromyxobacter
Deltaproteobacteria-Archangiaceae-Archangium
Deltaproteobacteria-Archangiaceae-Unclassified
Deltaproteobacteria-bacteriap25-Unclassified
Deltaproteobacteria-Bacteriovoracaceae-Bacteriovorax
Deltaproteobacteria-Bacteriovoracaceae-Peredibacter
Deltaproteobacteria-Bdellovibrionaceae-Bdellovibrio
Deltaproteobacteria-Bdellovibrionaceae-OM27_clade
Deltaproteobacteria-Blrii41-Unclassified
Deltaproteobacteria-Blfdi19-Unclassified
Deltaproteobacteria-Desulfarculaceae-Unclassified
Deltaproteobacteria-Geobacteraceae-Geobacter
Deltaproteobacteria-Haliangiaceae-Haliangium
Deltaproteobacteria-KD3-10-Unclassified
Deltaproteobacteria-mle1-27-Unclassified
Deltaproteobacteria-MSB-4B10-Unclassified
Deltaproteobacteria-Myxococcaceae-Unclassified
Deltaproteobacteria-Oligoflexaceae-Unclassified
Deltaproteobacteria-P3OB-42-Unclassified
Deltaproteobacteria-Phaselicystidaceae-Phaselicystis
Deltaproteobacteria-Polyangiaceae-Minicystis
Deltaproteobacteria-Polyangiaceae-Pajaroellobacter

Class-Family-Genus continued

- Deltaproteobacteria–Polyangiaceae–Unclassified
- Deltaproteobacteria–Sandaracinaceae–Sandaracinus
- Deltaproteobacteria–Sandaracinaceae–Unclassified
- Deltaproteobacteria–Unclassified–Unclassified
- Elusimicrobia–Unclassified–Unclassified
- Fibrobacteria–Fibrobacteraceae–possible genus 04
- Fibrobacteria–Fibrobacteraceae–Unclassified
- Fimbriimonadia–Fimbriimonadaceae–Fimbriimonas
- Fimbriimonadia–Fimbriimonadaceae–Unclassified
- Gammaproteobacteria–A21b–Unclassified
- Gammaproteobacteria–B1–7BS–Unclassified
- Gammaproteobacteria–Burkholderiaceae–Achromobacter
- Gammaproteobacteria–Burkholderiaceae–Aquincola
- Gammaproteobacteria–Burkholderiaceae–Azohydromonas
- Gammaproteobacteria–Burkholderiaceae–Burkholderia–Caballeronia–Paraburkholderia
- Gammaproteobacteria–Burkholderiaceae–Caenimonas
- Gammaproteobacteria–Burkholderiaceae–Cupriavidus
- Gammaproteobacteria–Burkholderiaceae–Duganella
- Gammaproteobacteria–Burkholderiaceae–Kinneretia
- Gammaproteobacteria–Burkholderiaceae–Massilia
- Gammaproteobacteria–Burkholderiaceae–Mitsuaria
- Gammaproteobacteria–Burkholderiaceae–Paucibacter
- Gammaproteobacteria–Burkholderiaceae–Piscinibacter
- Gammaproteobacteria–Burkholderiaceae–Pseudoduganella
- Gammaproteobacteria–Burkholderiaceae–Ralstonia
- Gammaproteobacteria–Burkholderiaceae–Ramlibacter
- Gammaproteobacteria–Burkholderiaceae–Roseateles
- Gammaproteobacteria–Burkholderiaceae–Unclassified
- Gammaproteobacteria–Diplorickettsiaceae–Aquicella
- Gammaproteobacteria–Diplorickettsiaceae–Unclassified
- Gammaproteobacteria–Enterobacteriaceae–Unclassified
- Gammaproteobacteria–Legionellaceae–Legionella
- Gammaproteobacteria–Methylophilaceae–Methylophilus
- Gammaproteobacteria–Moraxellaceae–Cavicella
- Gammaproteobacteria–Moraxellaceae–Unclassified
- Gammaproteobacteria–Nitrosomonadaceae–Ellin6067
- Gammaproteobacteria–Nitrosomonadaceae–MND1
- Gammaproteobacteria–Nitrosomonadaceae–Unclassified
- Gammaproteobacteria–Pseudomonadaceae–Pseudomonas
- Gammaproteobacteria–Rhodanobacteraceae–Dokdonella
- Gammaproteobacteria–Rhodanobacteraceae–Dyella
- Gammaproteobacteria–Rhodanobacteraceae–Frateuria
- Gammaproteobacteria–Rhodanobacteraceae–Fulvimonas
- Gammaproteobacteria–Rhodanobacteraceae–Luteibacter
- Gammaproteobacteria–Rhodanobacteraceae–Rhodanobacter
- Gammaproteobacteria–Rhodanobacteraceae–Tahibacter
- Gammaproteobacteria–Rhodanobacteraceae–Unclassified
- Gammaproteobacteria–Rhodocyclaceae–Unclassified
- Gammaproteobacteria–SC–I–84–Unclassified
- Gammaproteobacteria–Solimonadaceae–Alkanibacter
- Gammaproteobacteria–Solimonadaceae–Fontimonas
- Gammaproteobacteria–Solimonadaceae–Nevskia
- Gammaproteobacteria–Solimonadaceae–Panacagrimonas
- Gammaproteobacteria–Solimonadaceae–Polycyclovorans
- Gammaproteobacteria–Steroidobacteraceae–Povalibacter
- Gammaproteobacteria–Steroidobacteraceae–Steroidobacter
- Gammaproteobacteria–Steroidobacteraceae–Unclassified
- Gammaproteobacteria–TRA3–20–Unclassified
- Gammaproteobacteria–Unclassified–Unclassified
- Gammaproteobacteria–Unknown_Family–Acidibacter
- Gammaproteobacteria–Xanthomonadaceae–Arenimonas
- Gammaproteobacteria–Xanthomonadaceae–Luteimonas
- Gammaproteobacteria–Xanthomonadaceae–Lysobacter
- Gammaproteobacteria–Xanthomonadaceae–Thermomonas
- Gammaproteobacteria–Xanthomonadaceae–Unclassified
- Gemmatimonadetes–Gemmatimonadaceae–Gemmatimonas
- Gemmatimonadetes–Gemmatimonadaceae–Gemmatirosa
- Gemmatimonadetes–Gemmatimonadaceae–Unclassified
- Holophagae–Holophagaceae–Geothrix
- Holophagae–Unclassified–Unclassified
- Ignavibacteria–Unclassified–Unclassified
- Ktedonobacteria–Ktedonobacteraceae–HSB OF53–F07
- Ktedonobacteria–Ktedonobacteraceae–Ktedonobacter
- Ktedonobacteria–Ktedonobacteraceae–Unclassified
- Ktedonobacteria–Unclassified–Unclassified
- Longimicrobia–Longimicrobiaceae–Unclassified
- Melainabacteria–Unclassified–Unclassified
- NC10–Unclassified–Unclassified
- Nitrososphaeria–Nitrososphaeraceae–Candidatus Nitrocosmicus
- Nitrososphaeria–Nitrososphaeraceae–Candidatus Nitrososphaera
- Nitrososphaeria–Nitrososphaeraceae–Unclassified
- Nitrospira–Nitrospiraceae–Nitrospira
- Parcubacteria–Unclassified–Unclassified
- Phycisphaerae–CPla–3 termite group–Unclassified
- Phycisphaerae–Phycisphaeraceae–SM1A02
- Phycisphaerae–Phycisphaeraceae–Unclassified
- Phycisphaerae–Tepidisphaeraceae–Tepidisphaera
- Phycisphaerae–Tepidisphaeraceae–Unclassified
- Phycisphaerae–Unclassified–Unclassified
- Phycisphaerae–WD2101 soil group–Unclassified
- Planctomycetacia–Gemmataceae–Fimbriiglobus
- Planctomycetacia–Gemmataceae–Gemmata
- Planctomycetacia–Gemmataceae–Telmatocola
- Planctomycetacia–Gemmataceae–Unclassified
- Planctomycetacia–Gemmataceae–Zavarzinella
- Planctomycetacia–Isosphaeraceae–Singulisphaera
- Planctomycetacia–Pirellulaceae–Pirellula
- Planctomycetacia–Pirellulaceae–Unclassified
- Planctomycetacia–Rubinisphaeraceae–Planctomicrobium
- Planctomycetacia–Rubinisphaeraceae–SH–PL14
- Planctomycetacia–Schlesneriaceae–Schlesneria
- Planctomycetacia–Unclassified–Unclassified
- Saccharimonadia–Saccharimonadaceae–Unclassified
- Saccharimonadia–Unclassified–Unclassified
- Spirochaetia–Spirochaetaceae–Salinispira
- Spirochaetia–Spirochaetaceae–Spirochaeta 2
- Subgroup_6–Unknown Family–Vicinamibacter
- Thermoanaerobaculia–Thermoanaerobaculaceae–Subgroup 10
- Thermoleophilia–67–14–Unclassified
- Thermoleophilia–Gaiellaceae–Gaiella
- Thermoleophilia–Solirubrobacteraceae–Conexibacter
- Thermoleophilia–Solirubrobacteraceae–Parviterribacter
- Thermoleophilia–Solirubrobacteraceae–Solirubrobacter
- Thermoleophilia–Solirubrobacteraceae–Unclassified
- Thermoleophilia–Unclassified–Unclassified
- Verrucomicrobiae–Chthoniobacteraceae–Candidatus Udaeobacter
- Verrucomicrobiae–Chthoniobacteraceae–Chthoniobacter
- Verrucomicrobiae–Chthoniobacteraceae–LD29
- Verrucomicrobiae–Chthoniobacteraceae–Unclassified
- Verrucomicrobiae–Methylacidiphilaceae–Unclassified
- Verrucomicrobiae–Opitutaceae–Lacunisphaera
- Verrucomicrobiae–Opitutaceae–Opitutus
- Verrucomicrobiae–Opitutaceae–Unclassified
- Verrucomicrobiae–Pedosphaeraceae–ADurb.Bin063–1
- Verrucomicrobiae–Pedosphaeraceae–Pedosphaera
- Verrucomicrobiae–Pedosphaeraceae–Unclassified
- Verrucomicrobiae–Terrimicrobiaceae–Terrimicrobium
- Verrucomicrobiae–Verrucomicrobiaceae–Prosthecobacter
- Verrucomicrobiae–Verrucomicrobiaceae–Unclassified
- Verrucomicrobiae–Xiphinematobacteraceae–Candidatus Xiphinematobacter

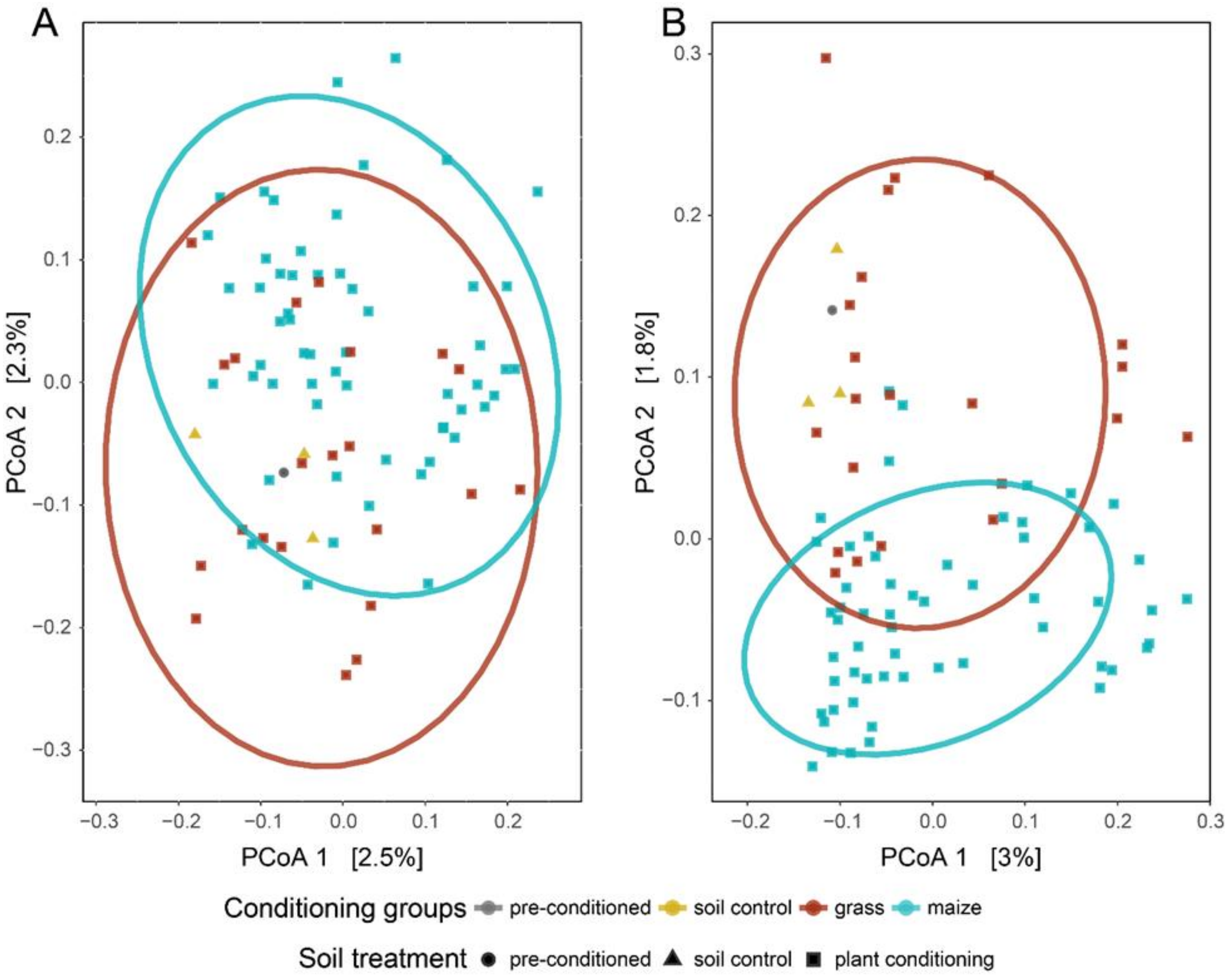

Figure S2. Variation in ASV microbial community composition between soil conditioning types. Principle coordinate analysis (PCoA) using a Bray-Curtis (abundance-weighted) (A) and Jaccard (presence-absence) (B) dissimilarity distance indices of amplicon sequence variants (ASVs) between soil conditioning treatments. Points correspond to samples from individual pots (square points) conditioned by prairie grasses (red) or maize genotypes (aqua), from pre-conditioned soil (gray circles), and from negative control soil (yellow triangles). Colors and symbols indicate conditioning functional groups and soil treatments, respectively. The soil treatments include pre-conditioned soil samples (grey circles) and unconditioned control pots that were not conditioned by plants (yellow triangles). The conditioning functional groups are indicated by square points and were colored so the prairie grass spp. group (*A. gerardii* and *T. dactyloides*) coordinated with a red-orange hue while the maize genotypes groups (*Z. mays* ssp, *parviglumis*, *Z. mays* ssp. *mays b58*, *B73-wt*, *B73-rth3*, and *HP301*) coordinated with a light blue hue. Ellipses represent the 95% confidence ellipse based on the standard deviation around the centroid for conditioning treatments with more than 5 biological replicates.

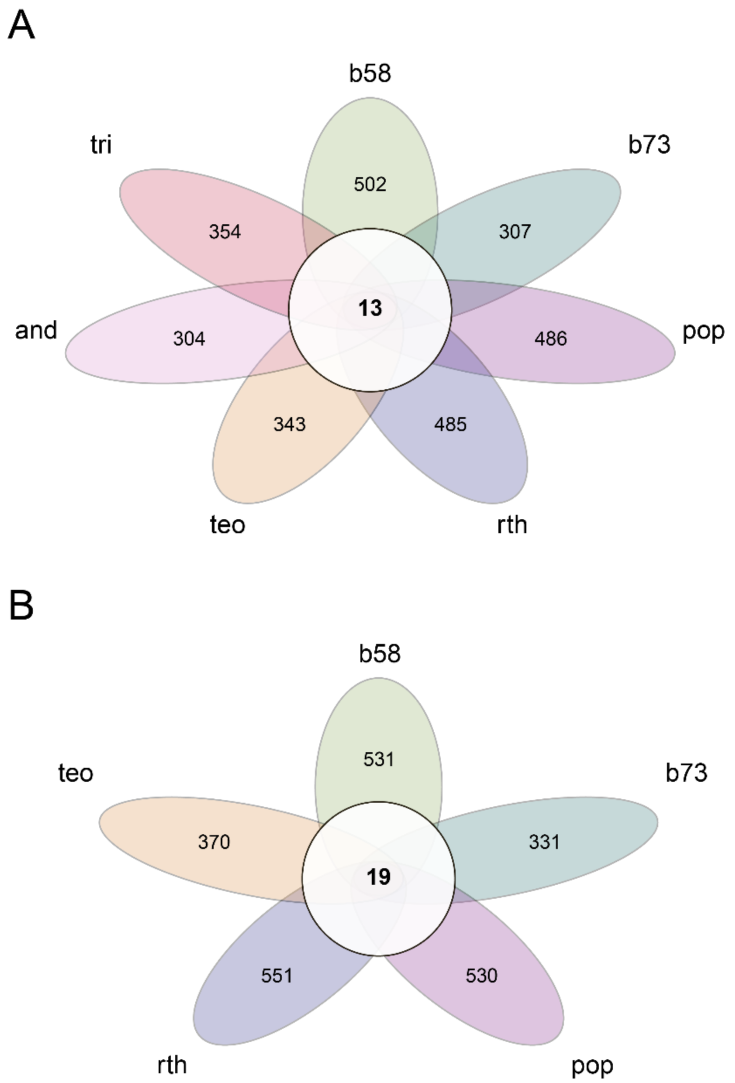

Figure S3. Shared and unique microbial taxa in maize and Poaceae rhizobiomes. Petal plots of amplicon sequence variants (ASV) of A) all conditioning genotypes included in this study (*A. gerardii*, *T. dactyloides, Z. mays* ssp, *parviglumis*, *Z. mays* ssp. *mays b58*, *B73-wt*, *B73-rth3*, and *HP301*) and B) the maize conditioning genotypes (*Z. mays* ssp, *parviglumis*, *Z. mays* ssp. *mays b58*, *B73-wt*, *B73-rth3*, and *HP301*). The number within each ellipse represents the number of ASVs unique to each conditioning genotype within each analysis, along with a centroid including the total shared ASVs across conditioning genotypes in each analysis. The microbiota of all rhizosphere conditioning genotype communities included 3592 ASVs and the maize conditioning genotype microbiota included 2890 ASVs.

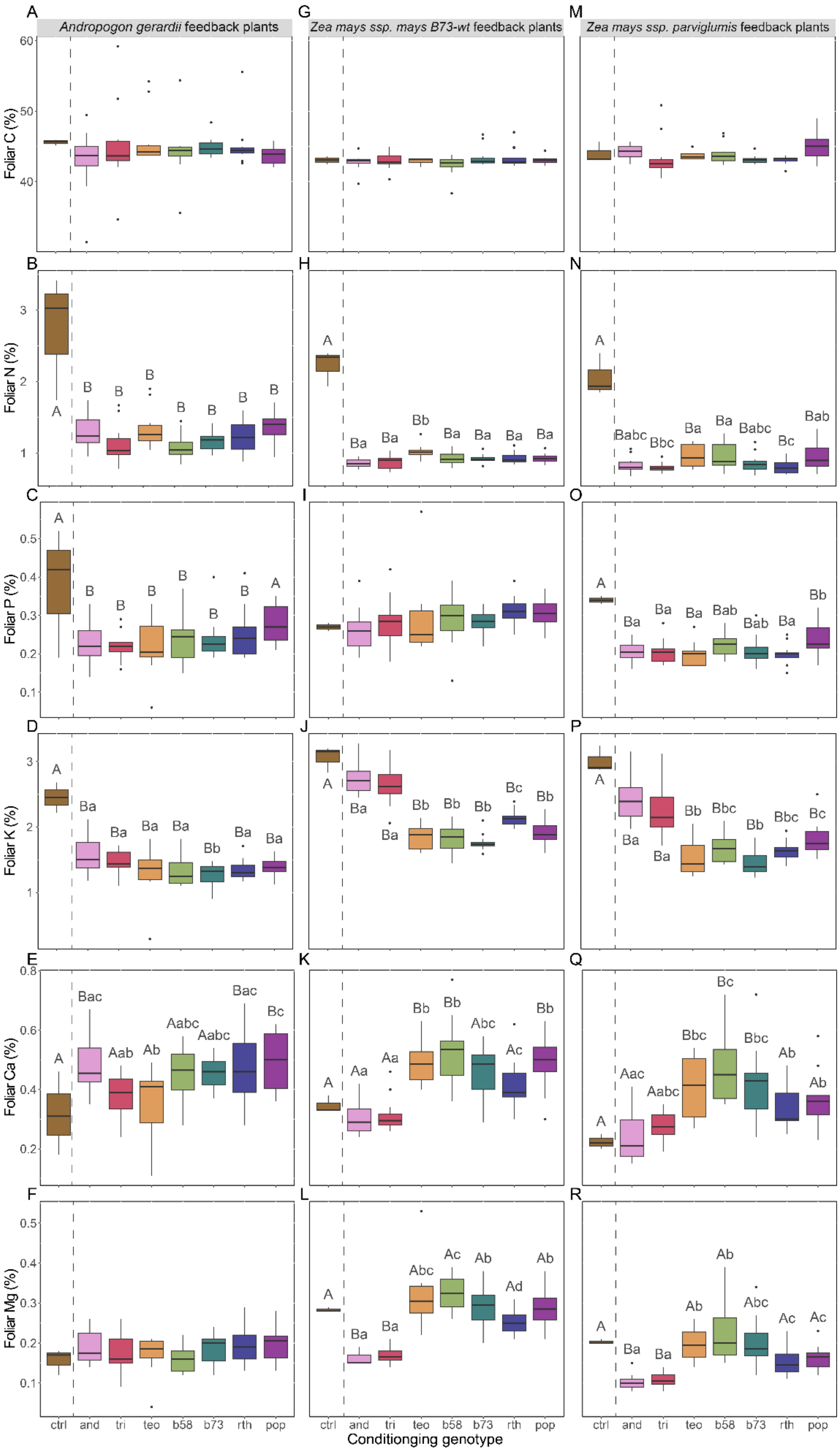

A
G
M
Andropogon gerardii feedback plants
Zea mays ssp. mays B73-wt feedback plants
Zea mays ssp. parviglumis feedback plants
Foliar C (%)
B
H
N
Foliar N (%)
C
I
O
Foliar P (%)
D
J
P
Foliar K (%)
E
K
Q
Foliar Ca (%)
F
L
R
Foliar Mg (%)
ctrl and tri teo b58 b73 rth pop
Conditionging genotype

Figure S4. Variation in foliar nutrient concentrations of feedback plants among conditioning genotypes and controls. Foliar nutrient concentrations were quantified for conditioning genotype effect using type III analysis of variance (ANOVAs) of separate linear mixed models for leaf carbon (A, G, and M), nitrogen (B, H, and N), phosphorus (C, I, and O), potassium (D, J, and P), calcium (E, K, and Q), and magnesium (F, L, and R) in *A. gerardii* (A-F), maize *B73-wt* (G-L), and teosinte feedback plants (M-R). Colors indicate conditioning genotypes and other soil treatments. The leftmost category includes soil samples from unconditioned control pots that were not conditioned by plants but did have feedback plants growing in them (brown, "ctrl"). Thus, the difference between the plant-conditioned versus the unconditioned soils reflects the effects of plant conditioning. The prairie grass species are pink (*Andropogon gerardii*; 'and') and red (*Tripsacum dactyloides*; 'tri'). Maize genotypes of *Z. mays ssp. parviglumis* are light orange (teosinte, 'teo') and the *Z. mays* ssp. *mays* genotypes are in cooler colors: *b58* is light green, *B73-wt* is dark green, *B73-rth3* ('rth') is dark blue, and *HP301* ('pop') is purple. Lowercase letters indicate significant differences between conditioning genotypes and uppercase letters indicate significant differences between conditioning and negative controls, from *post-hoc* pairwise comparisons using Benjamini-Hochberg correction. Refer to Table 3 for omnibus tests of conditioning genotype effects.

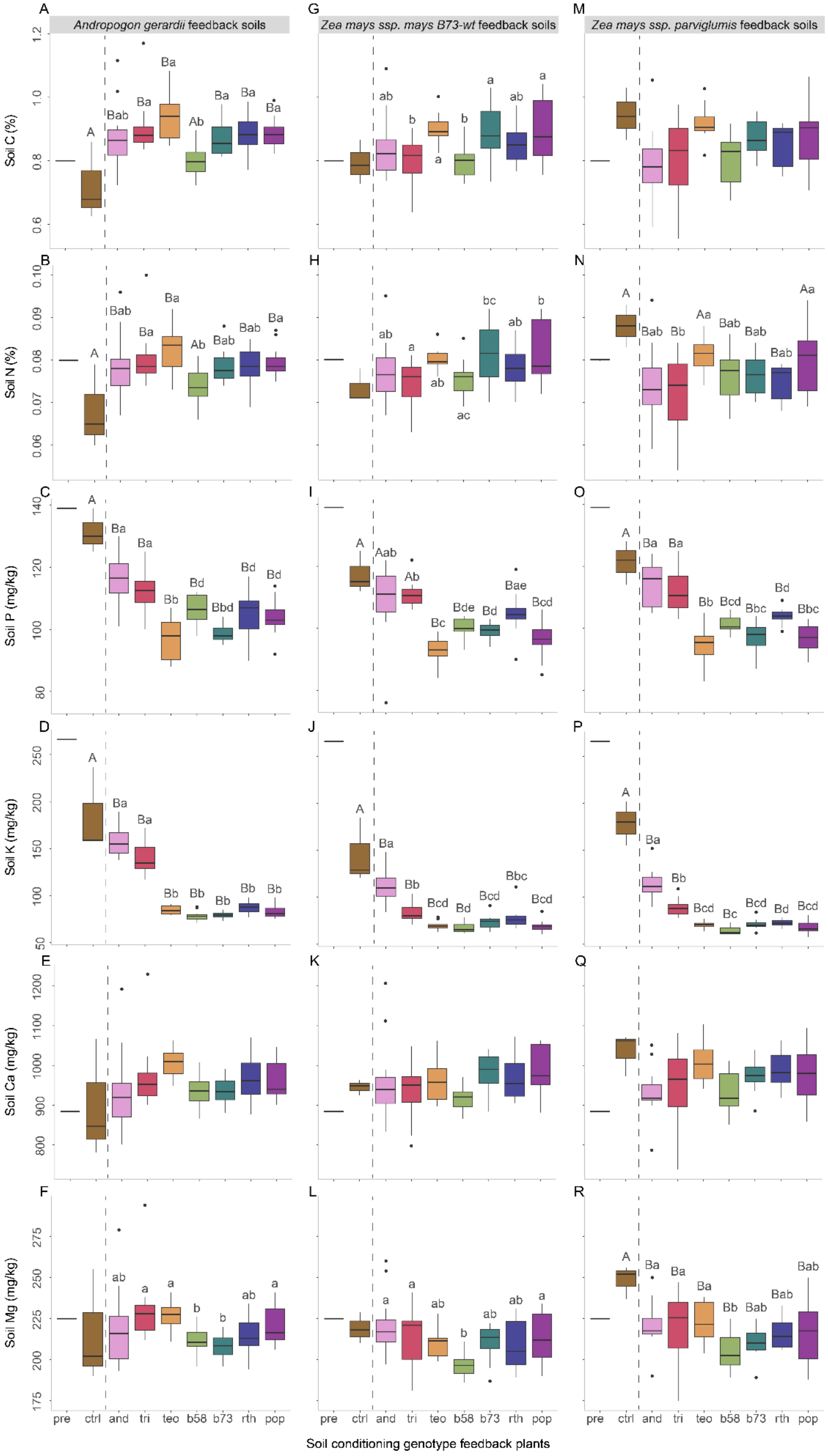
A
Andropogon gerardii feedback soils
G
Zea mays ssp. mays B73-wt feedback soils
M
Zea mays ssp. parviglumis feedback soils
Soil C (%)
B
H
N
Soil N (%)
C
I
O
Soil P (mg/kg)
D
J
P
Soil K (mg/kg)
E
K
Q
Soil Ca (mg/kg)
F
L
R
Soil Mg (mg/kg)
pre ctrl and tri teo b58 b73 rth pop
Soil conditioning genotype feedback plants

Figure S5. Variation in soil nutrient concentrations in pots of feedback plants among conditioning genotypes, preconditioned soils, and unconditioned control soils. Differences in total soil nutrient concentrations were quantified for conditioning genotype effect using type III analysis of variance (ANOVAs) of separate linear mixed models for soil carbon (A, G, and M), nitrogen (B, H, and N), phosphorus (C, I, and O), potassium (D, J, and P), calcium (E, K, and Q), and magnesium (F, L, and R) in *A. gerardii* (A-F), maize *B73-wt* (G-L), and teosinte feedback plants (M-R). The leftmost two categories are soil samples from a pre-conditioned soil sample (line, "pre") and from unconditioned control pots that were not conditioned by plants but did have feedback plants growing in them (brown, "ctrl"). Thus, the difference between the plant-conditioned versus the unconditioned soils reflects the effects of plant conditioning. Colors indicate conditioning genotypes and other soil conditioning treatments. The prairie grass species are pink (*Andropogon gerardii*; 'and') and red (*Tripsacum dactyloides*; 'tri'). Maize genotypes of *Z. mays ssp. parviglumis* are light orange (teosinte, 'teo') and the *Z. mays* ssp. *mays* genotypes are in cooler colors: *b58* is light green, *B73-wt* is dark green, *B73-rth3* ('rth') is dark blue, and *HP301* ('pop') is purple. Lowercase letters indicate significant differences between conditioning genotypes and uppercase letters indicate significant differences between conditioning and negative controls, from *post-hoc* pairwise comparisons using Benjamini-Hochberg correction. Refer to Table 3 for omnibus tests of conditioning genotype effects on final soil nutrient concentrations.

Appendix S3: Sample analysis code

The R programming code below provides the main code used for each research question.

```
#R version 4.1.2 (2021-11-01); RStudio version 2022-07-01+554

#Packages used for phenotypic analysis

library(vegan)

library(lme4)

library(lmerTest)

#Packages used for microbial analysis

library(tidyverse)

library(phyloseq)

library(factoextra)

library(ranacapa)

library(microbiome)

#packages used for visualization

library(ggplot2)


#datasets provided:

# filtered relative abundances of ASV table (microASV), metadata (microMET),
and taxonomic classifications (microTAX) in a phyloseq object:

#microASV shows relative abundances of each ASV as separate columns, a
biological replicate (pot)as rows, with numeric values as entries. Sample
dataset in comma delimited format:

#microMET has corresponding rows of biological replicates as rows, with each
metadata variable as entries in separate columns, including cond_gt
(conditioning genotype), cond_gt_group, soil_trt (including preconditioned and
negative control soils), with character class descriptions as entries

#microTAX is taxonomy table with ASVs as separate rows and phylum, kingdom,
class, order, family, genus, species in columns, with character class
descriptions as entries

microdat = phyloseq(microASV, microMET, microTAX)

# phenotypic traits data frame, including separate variables for plant
ecophysiological traits, foliar nutrient concentrations, and final soil
nutrient concentrations in columns, biological replicate (pot-level samples) as
rows, with numeric values as entries:

phenodat


#Q1 analysis: rhizosphere community structure
```

```
#taxanomic bar plots:

a <- microdat %>%

	tax_glom(taxrank = "Class",NArm=F) %>%

	transform_sample_counts(function(x) {x/sum(x)} ) %>%

	psmelt() %>%

	arrange(Class)

b = ggplot(a, aes(x = cond_gt, y = Abundance, fill = group)) +

	xlab("Conditioning genotype") +

	geom_bar(position = "fill", stat = "identity") +

	theme(axis.text.x = element_text(angle = 90, hjust = 1),panel.background
	= element_rect(fill = "white"))

b

#plot composition of rhizosphere conditioning genotype group, initials, and
controls:

c <- phyloseq::ordinate(microdat, method = "PCoA", distance = "jaccard", binary
= T)

d <- plot_ordination(microdat, c, color = "cond_gt_group", shape = "soil_trt")
+

	geom_point(size = 3, alpha = 0.8) +

	stat_ellipse(inherit.aes = TRUE, level = 0.8, size = 2, alpha = 0.8) +

	theme(#panel.grid = element_line(color = 'gray', linetype = 2, size =
0.1),

	panel.background = element_rect(color = 'black', fill = 'transparent'),

	legend.key = element_rect(fill = 'transparent'))

d

#perMANOVA analysis for conditioning genotype nested within conditioning
genotype group:

dat1 <- cbind(microASV, microMET

e <- adonis(microASV ~ (cond_gt_group/cond_gt),

	data = dat1, permutations=999, method = "jaccard", binary = T)

e$aov.tab

# alpha diversity and ANOVA:

sample_data(microdat)$alpha<- microbiome::alpha(microdat, index = "all")

alphadat <- data.frame(as(sample_dat(microdat), "matrix"))

alphadat $obsrich = as.numeric(alphadat$alpha.obsrich)

anova(lmer(obsrich~cond_gt + (1|bio_replicate), data = alphadat))

summary(lmer(obsrich~cond_gt + (1|bio_replicate), data = alphadat))
```

```
#shared ASVs:

mecodat<- phyloseq2meco(microdat) #microeco package file type conversion

fit <- meco_dataset$merge_samples(use_group = "cond_gt_group")

fit.venn <- trans_venn$new(f.it, ratio = "seqratio")

fit.venn$plot_venn()

fit2 <- mecodat$merge_samples(use_group = "cond_gt")

fit2_venn <- trans_venn$new(fit2)

fit_petal <- fit2_venn$plot_venn(petal_plot = TRUE, petal_center_size = 50,
petal_r = 1.5, petal_a = 3, petal_move_xy = 3.8)

#indicator analysis:

abund = microASV

gt = microMET$cond_gt

inv = multipatt(abund, gt, func = "r.g", control = how(nperm=1999))

summary(inv)

inv$sign


#Q2.1 and 2.2 analyses: conditioning gt /group influence on plant performance
and phenotypes

#see Dryad dataset link for more information on phenotypic trait calculations

#omnibus tests of each growth rate for each feedback genotype

landmet = subset.data.frame(dat_min2, feedback_sp=="and")

m1 = lmer( Total_Biomass_Growth ~ cond_ gt + (1| cond_gt), data=landmet)

summary(m2)

m2 = lmer( Total_Biomass_Growth ~ cond_ gt + (1|cond_gt_group:cond_gt),
data=landmet)

summary(m2) #can replace “m2” with “m1” to see results for separate model

anova(m2)

#phenotypic traits and foliar nutrient concentrations add a control for initial
biomass to models within each feedback genotype:

n1 = lmer(rlr ~ cond gt + initial_biomass + (1|cond_gt_group:cond_gt),
data=landmet)

n2 = lmer(rlr ~ cond gt + initial_biomass + (1|cond_gt_group:cond_gt),
data=landmet)

summary(n2)

anova(n2)

#for all omnibus tests:
```

```
p.adjust(p, method = "BH", n =23) # p = p value resulting from anova(m); n argument is number of multiple comparisons

#for significant p-adjusted values from omnibus tests:

pairwise.t.test(landmet$rlr, landmet$cond_gt, p.adjust.method = "BH", alternative = "two.sided")

o<- ggplot(landmet, aes(x = cond_gt, y = rlr,fill = cond_gt, )) +

	geom_boxplot(position=position_dodge(width=1), show.legend = F) +

	theme(axis.text.y = element_text(angle = 90 ), text=element_text(size=21), plot.background = element_rect(fill = "white"), panel.background = element_rect(fill = "white"), axis.line.x = element_line(color = "black"), axis.line.y = element_line(color = "black"))

o



#Q2.3 analysis: conditioning gt and feedback gt influence on final soil nutrient concentrations

#omnibus tests for each final soil nutrient for each feedback genotype:

anova(lmer(soil_perc_C ~cond_gt + initial_biomass + (1|bio_rep), data = phenodat[which(phenodat$feedback_gt == "b73"),]))

#subset for pairwise comparisons

j = subset.data.frame(phenodat, feedback_gt=="b73")

pairwise.t.test(j$soil_perc_C, j$cond_gt, p.adjust.method = "BH")

#NMDS plot of final soil nutrient concentrations across conditioning genotypes, split by feedback genotype

f = pheno_dat[,c("soil_perc_C", "soil_perc_N", "soil_P_mgperkg", "soil_K_mgperkg", "soil_Ca_mgperkg", "soil_Mg_mgperkg")] #subsetting soil nutrient concentrations

g = metaMDS(f, distance = "gower", k=4, trymax = 1000, scale = TRUE)

stressplot(g)

h<- cbind(phenodat[,c("feedback_ID", "feedback_gt", "cond_gt", "soil_trt", "cond_gt_only", "cond_gt_group")], data.frame(g$points) #adding metadata to points produced by NMDS

i<-ggplot(h, aes(x=MDS1, y=MDS4, col = cond_gt_only, shape = cond_gt_group))+

		geom_point(alpha = 0.8, size = 3) +

		facet_wrap(~feedback_gt) +

		stat_ellipse(linewidth = 2, inherit.aes = TRUE) +

		theme(panel.background = element_rect(color = 'black', 'transparent'),

		legend.key = element_rect(fill = 'transparent'))

i
```
